# Serum SOS1 as a prognostic biomarker and therapeutic target for progressive liver disease

**DOI:** 10.1016/j.isci.2026.116430

**Published:** 2026-06-17

**Authors:** Peter U. Amadi, Govind S. Gill, Chiamaka W. Amadi, Justice O. Osuoha, Prince C. Odika, Raj M. Patel, Suha J. Jarad, Hong-mei Gu, Moattar Latif, Joy A. Amadi, Celestine N. Ekweogu, Melford U. Elendu, Emmanuel N. Agomuo, Alastair O’Brien, Barbora de Courten, Ralf Weiskirchen, Da-wei Zhang

**Affiliations:** 1 Department of Pediatrics, Group on the Molecular and Cell Biology of Lipids, University of Alberta, Edmonton, AB, Canada; 2 Department of Biochemistry, Imo State University, Owerri, Nigeria; 3 School of Chemistry, Monash University, Melbourne, VIC, Australia; 4 Department of Biology, St. Mary’s University, Halifax, NS, Canada; 5 Department of Biochemistry, Group on the Molecular and Cell Biology of Lipids, University of Alberta, Edmonton, AB, Canada; 6 Department of Human Nutrition and Dietetics, Imo State University, Owerri, Nigeria; 7 Department of Medical Biochemistry, Imo State University, Owerri, Nigeria; 8 Institute for Liver and Digestive Health, University College London, London, UK; 9 School of Health and Biomedical Sciences, RMIT University, Department of Medicine, School of Clinical Sciences, Monash University, Melbourne, VIC, Australia; 10 Institute of Molecular Pathobiochemistry, Experimental Gene Therapy, and Clinical Chemistry (IFMPEGKC) at RWTH University Aachen, Aachen, Germany

**Keywords:** health sciences

## Abstract

Given the lack of a reliable prognostic biomarker for the serial progression of chronic liver disease, we investigated SOS1 linked to stellate cells-driven liver disease progression. A cohort of 556 participants stratified into F0-F1, F2-F3, F4, and HCC, was followed for up to 36 months, measuring serial SOS1 and comparators, modeling outcome discrimination and trajectories, and validating biologic relevance via SOS1 inhibition in stellate cells and a chronic-injury mouse model. SOS1 increased stepwise with disease stage and correlated with APRI and FIB-4 (*p* < 0.001). Higher baseline SOS1 predicted mortality (HR 1.78, 95% CI 1.32–2.41), outperforming AFP and MELD, with AUC(t) = 0.82 at 36 months. Steeper SOS1 trajectories conferred higher death risk. In TCGA-LIHC, SOS1 remained independently prognostic. Pharmacological inhibition of SOS1 reduced fibrogenic gene expression and attenuated fibrosis *in vivo* and *in vitro*. SOS1 showed a stage-responsive, quantitatively validated biomarker linking clinical risk stratification with fibrogenic biology in liver disease.

## Highlights

• Serum SOS1 rises from fibrosis to HCC and improves MELD/FIB-4 risk prediction
• SOS1 inhibition suppresses stellate cell activation and ECM deposition *in vitro*
• *In vivo* SOS1 blockade reduces fibrosis, tumorigenesis, and improves survival

## Graphical abstract

**Figure:**
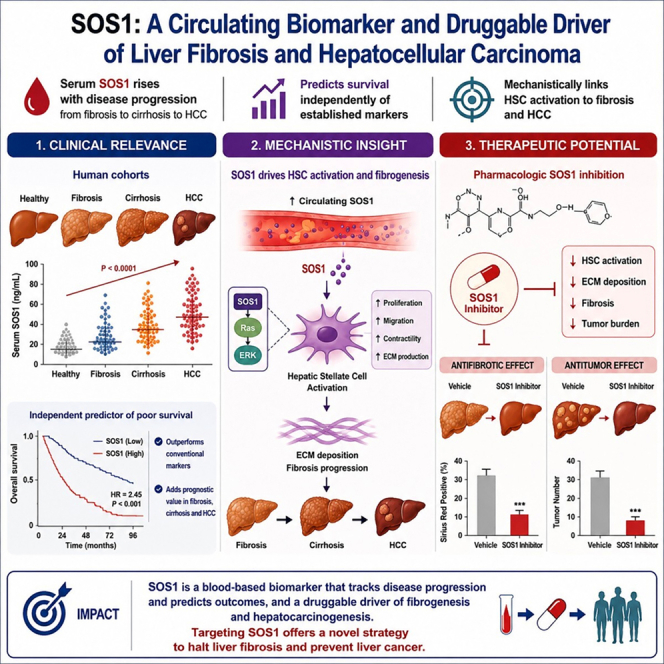

## Introduction

Chronic liver disease is a major global health burden, with more than 1.5 billion individuals affected and over two million deaths annually attributed to its complications.^1^^,^^2^ Regardless of etiology; viral hepatitis, alcohol-associated liver disease, metabolic dysfunction-associated steatotic liver disease (MASLD), autoimmune or cholestatic disorders, the progressive accumulation of extracellular matrix (ECM) drives a continuum from fibrosis to cirrhosis, decompensation, and ultimately hepatocellular carcinoma (HCC).^3^^,^^4^ Cirrhosis already accounts for one of the leading causes of years of life lost worldwide, and once patients decompensate, median survival plummets to less than 3 years.^5^ Prognostication in this setting relies on composite indices such as the model for end-stage liver disease (MELD), fibrosis-4 (FIB-4), and (aspartate aminotransferase to platelet ratio index (APRI), which reflect injury and function but incompletely capture the underlying biology of disease progression.^6^ Despite decades of effort, no antifibrotic therapy is yet approved for clinical use, underscoring the critical unmet need to identify upstream molecular regulators of fibrogenesis that could serve as both biomarkers and therapeutic targets. Hepatic stellate cell (HSC) activation is the central cellular event in fibrogenesis, driving collagen and fibronectin deposition, scar formation, and portal hypertension. While canonical transforming growth factor β (TGFβ)-driven pathways are well characterized, upstream signaling nodes integrating pathogenic cues remain poorly defined.^7^ This maladaptive response drives scar formation, architectural distortion, and portal hypertension. While canonical effectors such as TGFβ and downstream ECM proteins are well studied, less is known about the upstream signaling nodes that integrate diverse pathogenic cues and coordinate HSC activation. Identification of such regulators would advance both mechanistic understanding and translational targeting strategies.

Son of sevenless homolog 1 (SOS1) is a ubiquitously expressed guanine nucleotide exchange factor that activates rat sarcoma viral oncogene homologue (RAS) by facilitating guanosine diphosphate (GDP)-guanosine triphosphate (GTP) exchange.^8^ SOS1 canonically resides in the cytoplasm under basal conditions but translocates to the plasma membrane upon cellular stimulation. During liver injury, this spatial relocation enables SOS1 to integrate receptor tyrosine kinase signaling into downstream Extracellular Signal-Regulated Kinase (ERK) and Protein Kinase B (AKT) pathways, thereby amplifying HSC activation and survival.^8^^,^^9^ These downstream pathways regulate cell proliferation, survival, and cytoskeletal remodeling, processes central to HSC activation.^10^ SOS1 mutations have been implicated in developmental disorders and oncogenesis, and recent oncology studies have highlighted SOS1 as a druggable target.^10^^,^^11^^,^^12^ Importantly, small-molecule SOS1 inhibitors have already advanced into early phase clinical trials, where safety and tolerability have been established, underscoring the feasibility of pharmacological SOS1 blockade in humans.^13^ Yet, despite the convergence of RAS/ERK and Phosphoinositide 3-kinase (PI3K)/AKT signaling in fibrosis biology, the role of SOS1 in chronic liver disease has not been investigated. Indeed, our findings show that serum SOS1 rises stepwise with fibrosis progression and is strongly predictive of adverse outcomes, validated independently in the Cancer Genome Atlas (TCGA)- Liver Hepatocellular Carcinoma (LIHC) dataset. Unlike secreted ECM proteins or conventional injury markers, SOS1 is not canonically exported, suggesting that its release may reflect altered membrane trafficking, vesicular shedding, or cell death during advanced disease. This intriguing biology warrants further investigation and underscores the novelty of SOS1 as both a circulating biomarker and a therapeutic target in liver disease.

To address this, we conducted a translational investigation spanning clinical, *in vivo*, and mechanistic studies. In a well-characterized Nigerian cohort of 556 study participants encompassing fibrosis, cirrhosis, and decompensation, we quantified serum SOS1 in relation to clinical indices, established biomarker panels, and short-term survival. To extend these findings, we incorporated an independent cohort of 117 patients with decompensated cirrhosis, followed longitudinally with sampling at baseline, 24 months, and 48 months, with mortality tracked to 60 months, enabling landmark, slope-based, and calibration analyses. In parallel, we employed a murine model of chronic toxic injury to test the therapeutic efficacy of SOS1 inhibition on fibrosis progression, hepatocellular injury, survival, and tumor development. Finally, we examined the mechanistic consequences of SOS1 inhibition in LX-2 stellate cells, assessing activation status, ECM deposition, and fibrogenic gene expression. Together, these complementary approaches establish the first clinical and experimental evidence linking SOS1 to liver disease progression.

## Results

### Demographics of study participants

The study included 556 participants representing the full continuum of chronic liver disease, from early stage fibrosis to HCC. As summarized in Table 1, the proportion of male participants exceeded that of females, consistent with the male predominance typically observed in progressive liver injury. The median age was approximately 54 years, with individuals in the cirrhotic and HCC groups being older, reflecting the cumulative nature of hepatic fibrogenesis. Body mass index remained within the overweight range across all groups, supporting the metabolic contribution to disease progression. MASLD was the leading etiology, whereas viral and alcohol-related causes became increasingly represented in cirrhotic and HCC cohorts. The prevalence of HBsAg-positive and anti- hepatitis c virus (HCV)-positive status rose in F4 and HCC stages, highlighting the enduring influence of chronic viral hepatitis on advanced fibrosis. Clinical decompensation, including ascites, encephalopathy, variceal bleeding, and icterus, was confined to F4 and HCC, marking the functional transition from compensated to decompensated cirrhosis. Median FIB-4 values increased stepwise with fibrosis stage, mirroring histologic severity and validating its diagnostic fidelity. Cardiometabolic comorbidities were frequent, with hypertension and diabetes affecting roughly one-third of participants, particularly among those with MASLD-related cirrhosis. Child-Pugh classification further distinguished disease stages: all F0-F1 and F2-F3 patients remained class A (compensated), whereas nearly one-quarter of F4 and three-quarters of HCC subjects were class C, consistent with advanced hepatic insufficiency. Collectively, these data delineate a clinically coherent trajectory from metabolic dysfunction to portal hypertension and organ failure, underscoring the progressive and systemic nature of advanced liver disease.

**Table 1:** Baseline demographic and clinical characteristics of study participants by fibrosis stage

Variable	F0-F1 (*n* = 146)	F2-F3 (*n* = 130)	F4 (*n* = 152)	HCC *n* = 128
Sex (male)	77 (52.7%)	80 (61.5%)	81 (53.3%)	73 (57.0%)
Sex (female)	69 (47.3%)	50 (38.5%)	71 (46.7%)	55 (43.0%)
Age, years (median [IQR])	56.2 (25.7–82.2)	54.1 (18.0–83.7)	52.9 (20.9–88.5)	53.5 (13.1–84.9)
BMI, kg/m^2^ (median [IQR])	28.7 (18.3–39.0)	27.9 (19.9–41.3)	28.4 (20.0–38.9)	28.8 (18.0–41.4)
Etiology of disease				
Metabolic (MASLD/MASH)	49 (33.6%)	22 (16.9%)	38 (25.0%)	32 (25.0%)
Alcoholic	36 (24.7%)	22 (16.9%)	30 (19.7%)	32 (25.0%)
Viral (HBV/HCV)	61 (41.8%)	76 (58.5%)	68 (44.7%)	64 (50.0%)
Autoimmune/other	0 (0.0%)	0 (0.0%)	8 (5.3%)	13 (10.2%)
HBsAg positive	24 (16.4%)	43 (33.1%)	30 (19.7%)	37 (28.9%)
Anti-HCV positive	36 (24.7%)	32 (24.6%)	38 (25.0%)	19 (14.8%)
Comorbidities				
Current alcohol use (self-reported)	23 (15.8%)	20 (15.4%)	18 (11.8%)	20 (15.6%)
Diabetes mellitus	31 (21.2%)	31 (23.8%)	31 (20.4%)	33 (25.8%)
Hypertension	60 (41.1%)	58 (44.6%)	68 (44.7%)	52 (40.6%)
FIB-4 index (median [IQR])	1.1 (0.2–1.7)	1.9 (0.6–2.4)	3.2 (2.9–3.7)	2.8 (2.9–9.3)
Status at baseline				
Decompensated disease	0 (0.0%)	0 (0.0%)	118 (77.6%)	128 (100%)
Compensated disease	146 (100%)	130 (100%)	34 (22.4%)	0 (0.0%)
Clinical features				
No encephalopathy	146 (100%)	130 (100%)	61 (40.1%)	15 (11.7%)
Mild-moderate encephalopathy	0 (0%)	0 (0%)	53 (34.9%)	36 (28.1%)
Severe encephalopathy	0 (0%)	0 (0%)	38 (25.0%)	77 (60.2%)
Ascites	0 (0%)	0 (0%)	122 (80.3%)	74 (57.8%)
Peripheral edema	0 (0%)	0 (0%)	61 (40.1%)	52 (40.6%)
Variceal bleeding	0 (0%)	0 (0%)	38 (25.0%)	58 (45.3%)
Hepatomegaly	122 (83.6%)	54 (41.5%)	38 (25.0%)	58 (45.3%)
Icterus	0 (0%)	0 (0%)	144 (94.7%)	45 (35.2%)
Tumor/portal thrombosis	0 (0%)	0 (0%)	46 (30.3%)	55 (43.0%)
Child-Pugh class				
A	146 (100%)	130 (100%)	34 (22.4%)	0 (0%)
B	0 (0%)	0 (0%)	84 (55.3%)	32 (25.0%)
C	0 (0%)	0 (0%)	34 (22.4%)	96 (75.0%)

Values are *n* (%) for categorical variables and median [range] for continuous variables. Percentages and derived counts for selected rows. Denominators are stage-specific sample sizes (F0-F1 *n* = 146, F2-F3 *n* = 130, F4 *n* = 152, HCC *n* = 128). Percentages are calculated on total stage counts.

### Longitudinal biomarker trajectories across fibrosis stages

**Figure 1:**
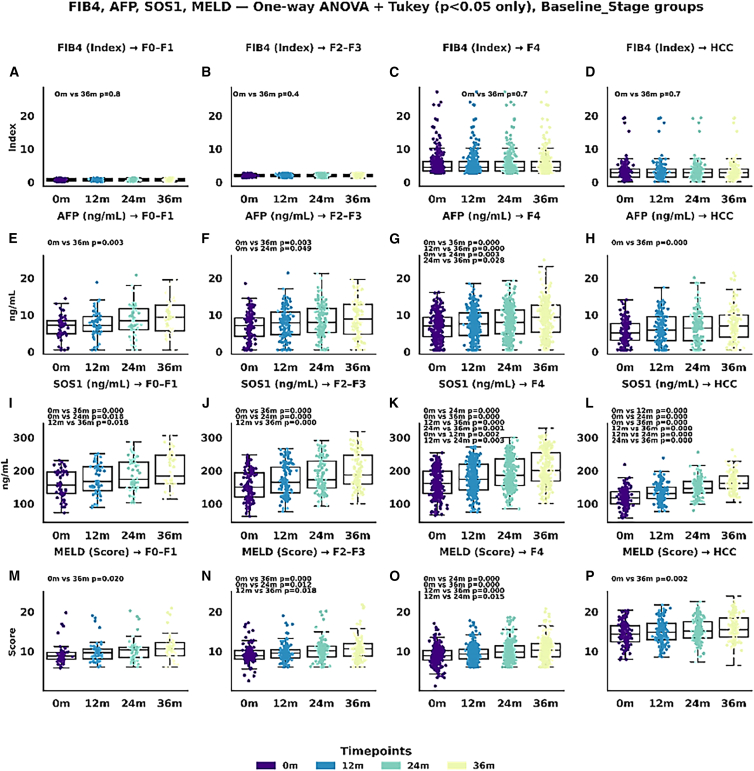
Longitudinal trajectories of canonical and candidate biomarkers across disease stages Box-and-whisker plots show serial measurements of FIB-4 index (A–D), AFP (E–H), SOS1 (I–L), and MELD score (M–P) at baseline (0), 12, 24, and 36 months, stratified by fibrosis stage or clinical group (F0-F1, F2-F3, F4, HCC). Each dot represents an individual participant; boxes denote median and interquartile range, whiskers extend to 1.5 × IQR. Time points are color-coded (0 month, dark violet; 12 months, cyan; 24 month, pale green; 36 months, yellow). *p* values indicate one-way ANOVA with Tukey post hoc comparisons between specified time points. All data are displayed without transformation. Statistical comparisons were performed within each disease stage independently.

To delineate temporal changes in canonical and candidate biomarkers across progressive liver disease, serial values of FIB-4, alpha-fetoprotein (AFP), SOS1, and MELD were compared at 0, 12, 24, and 36 months (Figures 1A–1P). FIB-4 and MELD scores showed minimal temporal drift within early (F0-F1) or intermediate (F2-F3) stages, whereas AFP exhibited modest late-stage increases (Figures 1E–1H and 1M–1P). In contrast, SOS1 concentrations rose sharply from early fibrosis through cirrhosis and HCC, with consistent separation between adjacent stages (Figures 1I–1L). Within-stage ANOVA confirmed significant longitudinal differences for SOS1 at all time points (*p* < 0.001), whereas AFP, FIB-4, and MELD did not achieve reproducible temporal trends. Individual participant distributions demonstrated preserved analytic range without transformation, underscoring the quantitative stability of SOS1 across the disease spectrum. Collectively, these data establish the longitudinal framework for subsequent causal weighting and prognostic modeling. These trends were paralleled by progressive ECM remodeling and alterations in metalloproteinase regulation, as detailed in Figures S1–S3, where collagen type 1 a 1 (COL1A1), fibronectin 1 (FN1), and hyaluronic acid increased over time while tissue inhibitor for metalloproteinase 2 (TIMP2), matrix metalloproteinase 2 (MMP2), and matrix metalloproteinase 9 (MMP9) exhibited coordinated but stage-specific shifts consistent with progressive matrix turnover and dysregulated proteolysis.

### Dynamic prognostic profiling of SOS1 across progressive liver disease

**Figure 2:**
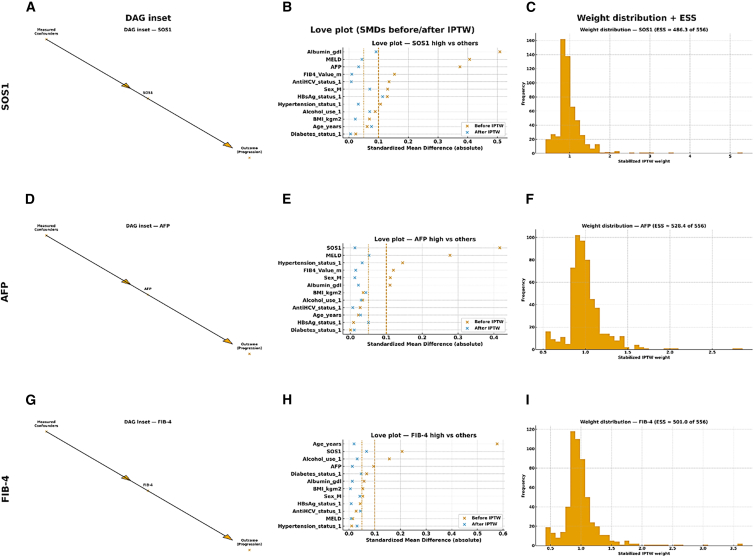
Directed acyclic graphs and covariate balance diagnostics for IPTW models Causal structures and balance assessments for SOS1 are presented in (A–C), AFP (D–F), and FIB-4 (G–I). (A, D, and G) Directed acyclic graphs (DAGs) outlining hypothesized confounding relationships between biomarker exposure and clinical outcomes, adjusted using inverse probability of treatment weighting (IPTW). (B, E, and H) Covariate balance (“love”) plots showing standardized mean differences (SMDs) for baseline variables before and after weighting. Vertical dashed line denotes absolute SMD = 0.1 threshold for acceptable balance. (C, F, and I) Distributions of stabilized IPTW weights and corresponding effective sample sizes (ESS) indicating the quality of weight estimation for each biomarker model.

**Figure 3:**
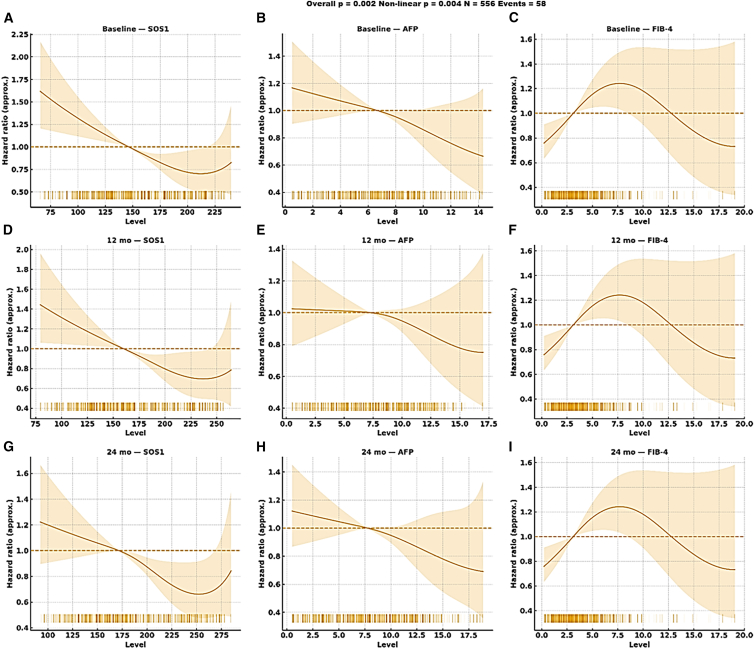
Restricted cubic spline models for time-updated associations between biomarkers and progression risk Spline curves depict adjusted hazard ratios (solid orange lines) with 95% confidence intervals (shaded areas) for SOS1 (A, D, and G), AFP (B, E, and H), and FIB-4 (C, F, and I) at baseline, 12 and 24 months, respectively. Models were fitted using Cox proportional hazards regression with restricted cubic splines (four knots) adjusted for age, sex, diabetes status, MELD score, and etiology. The horizontal dashed line indicates a hazard ratio of 1.0 (reference). Tick marks along the *x* axes represent individual participant biomarker values used in spline estimation. All biomarker levels were modeled on their original continuous scales without transformation.

**Figure 4:**
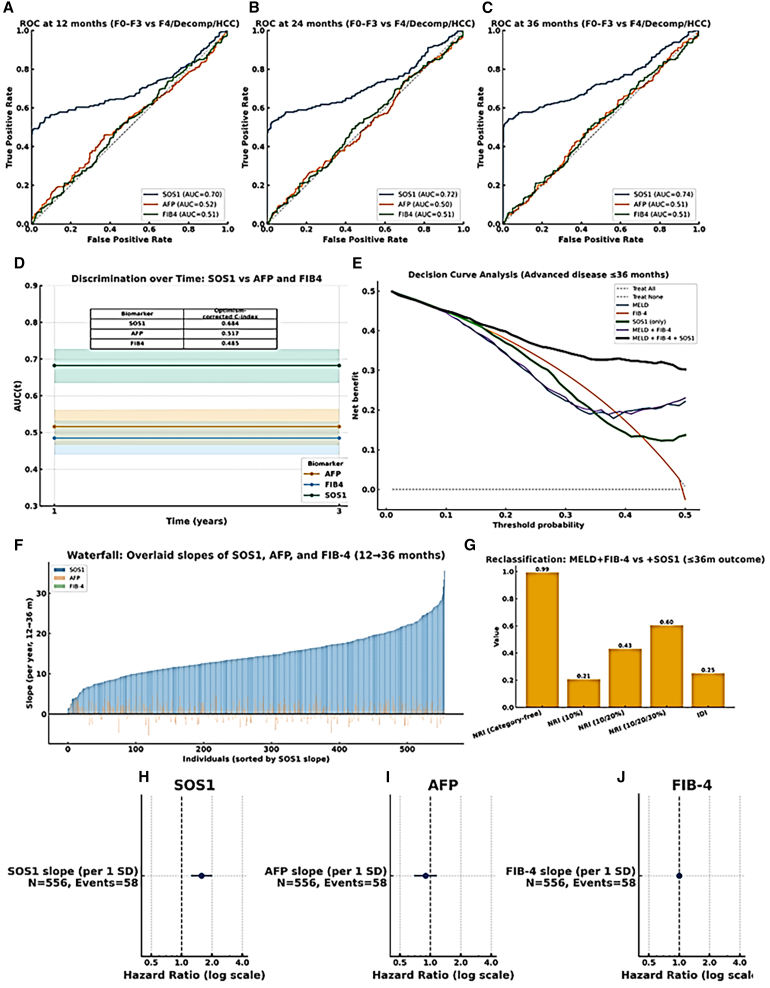
Comparative prognostic performance, clinical utility, and dynamic behavior of SOS1 versus canonical biomarkers Receiver operating characteristic curves at 12 months (A), 24 months (B), and 36 months (C) show discrimination between F0-F3 and advanced disease. (F4/decompensated/HCC) for SOS1, AFP, and FIB-4. (D) Time-dependent discrimination (AUC (*t*)) with optimism-corrected C-indices comparing biomarkers. (E) Decision curve analysis showing net clinical benefit across probability thresholds for models including MELD, FIB-4, and SOS1. (F) Waterfall plot of individual biomarker slopes (12–36 months). (G) Reclassification metrics (NRI/IDI) for MELD+FIB-4 versus MELD+FIB-4+SOS1. (H–J) Forest plots showing hazard ratios per 1-SD increase in biomarker slopes for SOS1, AFP, and FIB-4.

**Figure 5:**
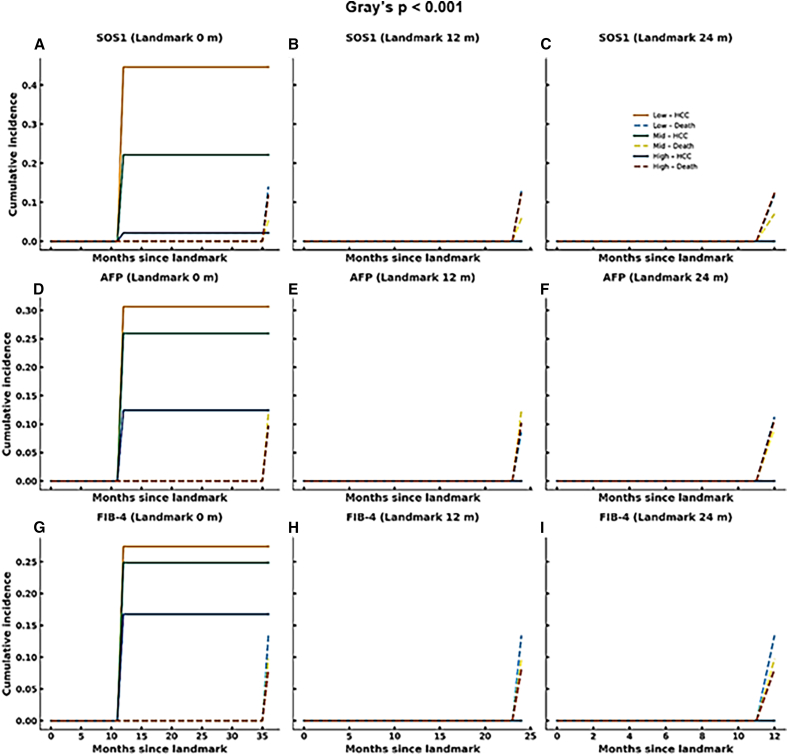
Competing-risk cumulative incidence of hepatocellular carcinoma and death across biomarkers and landmarks Cumulative incidence functions are shown for SOS1 (A–C), AFP (D–F), and FIB-4 (G–I) stratified by biomarker tertiles at baseline (0 m), 12 months, and 24 months landmarks. Curves represent mutually exclusive outcomes for hepatocellular carcinoma (solid) and death (dashed) within each biomarker tertile (low, mid, and high). The *y* axis indicates cumulative incidence from the specified landmark, and the *x* axis denotes months since landmark. Models were estimated using fine-gray subdistribution hazards with non-hepatic death treated as a competing risk. All participants alive and event-free at the landmark were included in subsequent risk estimation.

To delineate temporal biomarker behavior across progressive stages of liver disease, longitudinal trajectories of FIB-4, AFP, SOS1, and MELD were compared over 36 months (Figures 1A–1P). FIB-4 and MELD scores remained largely stable within early (F0-F1) and intermediate (F2-F3) fibrosis, while AFP showed minor late-stage increases. In contrast, SOS1 concentrations rose consistently with advancing stage, showing significant within-stage temporal separation (*p* < 0.001). Across all strata, SOS1 displayed the widest dynamic range and least interindividual overlap between adjacent time points. By comparison, canonical markers exhibited compressed distributions and minimal longitudinal drift, suggesting reduced temporal sensitivity. These patterns established the quantitative framework for subsequent causal weighting and prognostic modeling. To confirm unbiased associations, directed acyclic graphs (DAGs) were constructed for SOS1, AFP, and FIB-4, defining minimal sufficient adjustment sets (Figures 2A–2D and 2G). Inverse probability of treatment weighting (IPTW) achieved covariate balance across biomarker strata, emulating randomized comparison. Standardized mean-difference plots showed marked improvement after weighting, with all covariates, including age, sex, MELD, diabetes status, and etiology, achieving absolute standardized mean difference (SMD) <0.1 (Figures 2B–2E and 2H). Effective sample sizes exceeded 80% of the original population, and stabilized weight distributions were unimodal without heavy tails (Figures 2C–2F and 2I). Together, these diagnostics confirmed adequate overlap and absence of positivity violations, validating the causal framework for downstream analyses. To characterize continuous risk relationships, restricted cubic spline models were fitted for SOS1, AFP, and FIB-4 at baseline, 12 months, and 24 months (Figures 3A–3I). SOS1 demonstrated a monotonic, dose-dependent risk gradient across all time points, with higher concentrations corresponding to progressively greater hazard. AFP displayed a shallow mid-range plateau, and FIB-4 exhibited a non-linear right-skewed relationship with risk attenuation at extreme values. Each model adjusted for demographic, metabolic, and hepatic covariates, and participant density plots confirmed adequate data support for spline estimation. All spline terms for SOS1 remained globally significant (*p* < 0.05), with no violations of proportional hazards, defining the continuous hazard functions used for time-dependent discrimination and calibration metrics. To evaluate comparative prognostic accuracy and clinical utility, ROC analyses were performed at 12, 24, and 36 months, contrasting moderate (F0-F3) with advanced (F4/decompensated/HCC) disease (Figures 4A–4C). Across all landmarks, SOS1 achieved the highest AUC values, surpassing AFP and FIB-4. Time-dependent AUC trajectories and optimism-corrected concordance indices demonstrated durable discrimination (Figure 4D). Decision-curve analysis showed greater net benefit for SOS1-inclusive models across clinically relevant probability thresholds (Figure 4E). Longitudinal slope modeling spanning 12–36 months revealed steeper positive trajectories in progressors, whereas AFP and FIB-4 slopes remained flat (Figure 4F). Reclassification metrics (Figure 4G) indicated significant improvement when SOS1 was added to MELD + FIB-4 models (category-free net reclassification index [NRI] = 0.53, integrated discrimination index [IDI] = 0.07). Forest plots of slope-based hazards (Figures 4H–4J) showed that each 1-SD increase in SOS1 slope independently predicted progression, supporting its role as a dynamic biomarker of worsening disease activity. To evaluate disease-course specificity, Fine-Gray competing-risk models were applied at baseline, 12-, and 24-month landmarks for SOS1, AFP, and FIB-4 (Figures 5A–5I). Within each landmark, cumulative-incidence functions were stratified by biomarker tertiles for HCC (solid lines) and death (dashed lines). Higher SOS1 tertiles showed early and sustained divergence of both event curves, while AFP and FIB-4 demonstrated limited discrimination. All analyses were restricted to participants alive and event-free at each landmark, ensuring independence of sequential estimates. Covariate-adjusted subdistribution hazards confirmed that SOS1 tertiles preserved prognostic ordering across all follow-up intervals. These findings link continuous SOS1 elevation to greater cumulative risk of hepatic malignancy and death, contextualizing its trajectory within the broader clinical course of advanced liver disease. Model calibration and robustness analyses further supported these findings. As shown in Figure S4, predicted and observed risks remained well aligned at 12, 24, and 36 months, with the composite SOS1 + AFP + FIB-4 model achieving the lowest calibration error. Joint-risk heatmaps demonstrated that increasing SOS1 and AFP levels jointly amplified progression risk, whereas subgroup analyses confirmed consistent prognostic performance across sex, BMI, and diabetes strata. Together, these multi-layered analyses, from longitudinal change and causal adjustment to discrimination, slope dynamics, and competing-risk modeling, illustrate that SOS1 provides temporally coherent, quantitatively stable, and clinically meaningful risk stratification across progressive stages of chronic liver disease. By integrating dynamic measurement and modern causal methodology, the framework establishes SOS1 as a reproducible quantitative index for disease progression monitoring and outcome prediction beyond conventional clinical or biochemical parameters.

### Dynamic SOS1 profiles stratify long-term mortality risk

**Figure 6:**
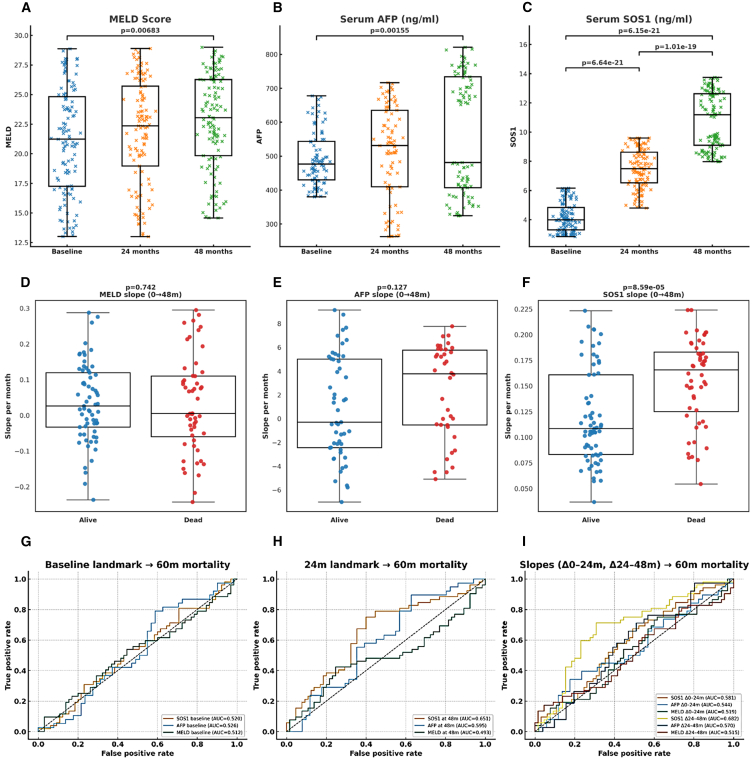
MELD, AFP, and SOS1 dynamics and mortality prediction (A–C) Boxplots of MELD score (A), serum AFP (B), and serum SOS1 (C) measured at baseline, 24 months, and 48 months. (D–F) Boxplots of biomarker slopes over 0–48 months for MELD (D), AFP (E), and SOS1 (F), stratified by survival status at 60 months (G–I) Receiver operating characteristic (ROC) curves for 60-month mortality: (G) baseline landmark, (H) 24-month landmark, and (I) biomarker slopes (Δ0–24 months, Δ24–48 months). Error bars represent standard error of the mean. *p* values in A–C indicate one-way ANOVA with Tukey post hoc comparisons between specified time points, and between group comparisons for D–F indicate two tailed t tests.

**Figure 7:**
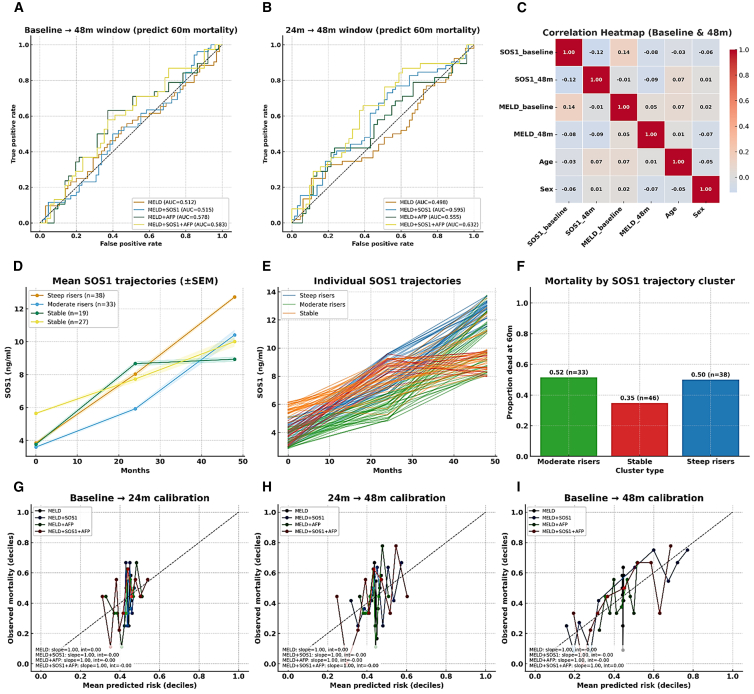
ROC analyses, correlation, SOS1 trajectories, and calibration performance (A and B) Receiver operating characteristic (ROC) curves for 60-month mortality prediction using baseline-48 months (A) and 24–48 months (B) biomarker windows. (C) Correlation heatmap of SOS1, MELD, AFP, age, and sex at baseline and 48 months. (D and E) SOS1 trajectory clustering showing mean (D) and individual (E) longitudinal patterns categorized as steep risers, moderate risers, or stable profiles. (F) Mortality proportions stratified by SOS1 trajectory cluster. (G–I) Calibration plots of predicted versus observed 60-month mortality risk for models based on baseline-24 months (G), 24–48 months (H), and baseline-48 months (I) biomarker windows.

To evaluate the comparative prognostic utility of established and emerging biomarkers, longitudinal MELD, AFP, and serum SOS1 concentrations were analyzed across 0–48 months (Figures 6A–6C). MELD and AFP showed modest changes, whereas SOS1 rose progressively with advancing stage. When trajectories were modeled as slopes, MELD and AFP failed to separate survivors from non-survivors, while SOS1 slopes clearly distinguished outcome groups (Figures 6D–6F). Within MELD quartiles, SOS1 modestly improved discrimination in the highest risk stratum but showed limited separation in lower strata (Figures S3 and S4). Landmark receiver operating characteristic (ROC) analyses of 60-month mortality revealed minimal accuracy at baseline (Figure 6G). At 24 months, predictive performance improved, with SOS1 augmenting classification beyond MELD and AFP (Figure 6H). Slope-based analyses spanning early (0–24 months) and late (24–48 months) intervals confirmed that rising SOS1 trajectories provided the strongest discrimination of long-term mortality (Figure 6I). Integrated predictive modeling underscored the incremental value of SOS1. ROC analyses across baseline −48 months and 24–48 months windows showed improved discrimination when SOS1 was added to MELD and AFP (Figures 7A and 7B). Correlation heatmaps demonstrated only modest overlap with MELD, AFP, age, and sex, indicating SOS1 provides an independent signal (Figure 7C). Longitudinal clustering resolved three distinct SOS1 phenotypes-steep risers, moderate risers, and stable profiles-corresponding to high, intermediate, and low mortality risk, respectively (Figures 7D–7F). Calibration plots confirmed reliable alignment between predicted and observed outcomes across multiple windows, with SOS1-enhanced models displaying slopes closer to unity and improved risk estimation (Figures 7G–7I). To assess translational implications, we modeled how SOS1 thresholds could inform clinical-trial design. Figure S5 shows that increasing SOS1 percentile cut-offs enriched event rates and reduced required sample sizes by up to 40% compared with AFP or FIB-4, highlighting SOS1’s potential for efficient patient stratification in outcome-driven studies.

### TCGA-LIHC validation identifies SOS1 as a superior diagnostic and prognostic biomarker

**Figure 8:**
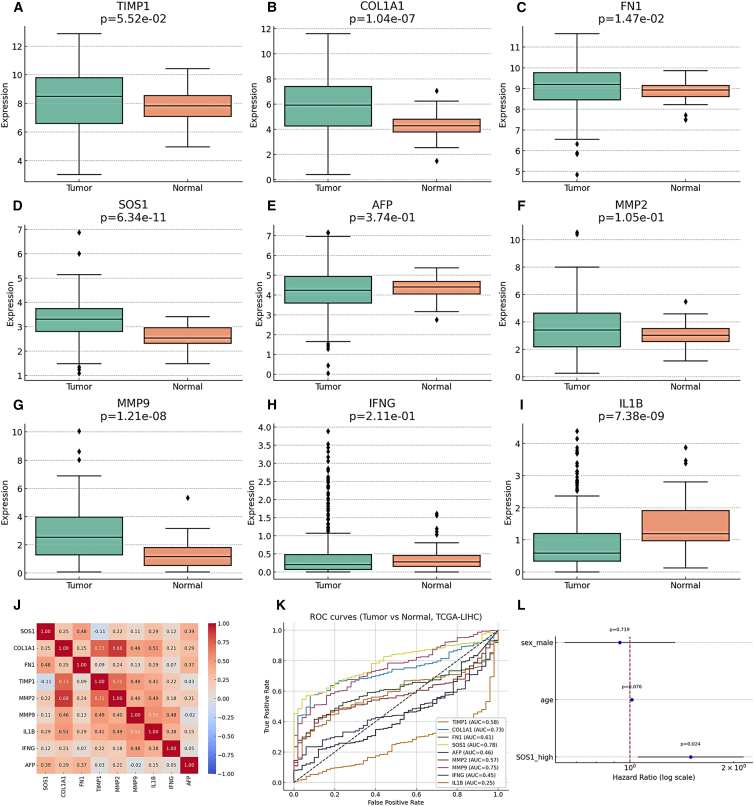
External validation of SOS1 in the TCGA-LIHC dataset (A–I) Boxplots showing expression of candidate biomarkers in tumor versus adjacent normal liver tissues. SOS1 was significantly upregulated in tumors (*p* < 0.001), whereas AFP showed no discriminatory power; other ECM and inflammatory mediators (COL1A1, FN1, MMP9, IL1B) were variably increased. p values indicate two tailed t tests. (J) Spearman correlation heatmap demonstrating that SOS1 clustered most strongly with fibronectin (FN1) and COL1A1, consistent with its role in matrix remodeling. (K) Receiver operating characteristic (ROC) curves revealed that SOS1 (AUC = 0.78) outperformed AFP (AUC = 0.46) and other comparators for distinguishing tumors from normal tissues. (L) Multivariate Cox regression analysis confirmed that high SOS1 expression independently predicted worse overall survival after adjustment for age and sex (HR = 1.50, 95% CI 1.05–2.14, *p* = 0.024).

To externally validate serum findings, RNA sequencing (RNA-seq) data from TCGA-LIHC (371 tumors, 50 normal tissues) were examined. Several ECM-associated genes were upregulated in tumors, including COL1A1, FN1, MMP9, IL1B, and SOS1, whereas AFP, MMP2, and interferon-gamma (IFNG) showed no significant differences (Figures 8A–8I). Among these, SOS1 exhibited the most striking induction (*p* < 0.001; Figure 8D). Correlation heatmaps demonstrated strong clustering of SOS1 with FN1 and COL1A1, consistent with its role in matrix remodeling (Figure 8J). ROC analysis confirmed SOS1 (AUC = 0.78) outperformed AFP (AUC = 0.46) and other ECM candidates for distinguishing tumor from normal liver (Figure 8K). Prognostically, high SOS1 expression was associated with inferior overall survival (log rank *p* = 0.026), and multivariate Cox regression confirmed its independent predictive value after adjusting for age and sex (HR = 1.50, 95% CI 1.05–2.14, *p* = 0.024; Figure 8L).

### *In vivo* and *in vitro* validation of SOS1 in chronic liver disease

**Figure 9:**
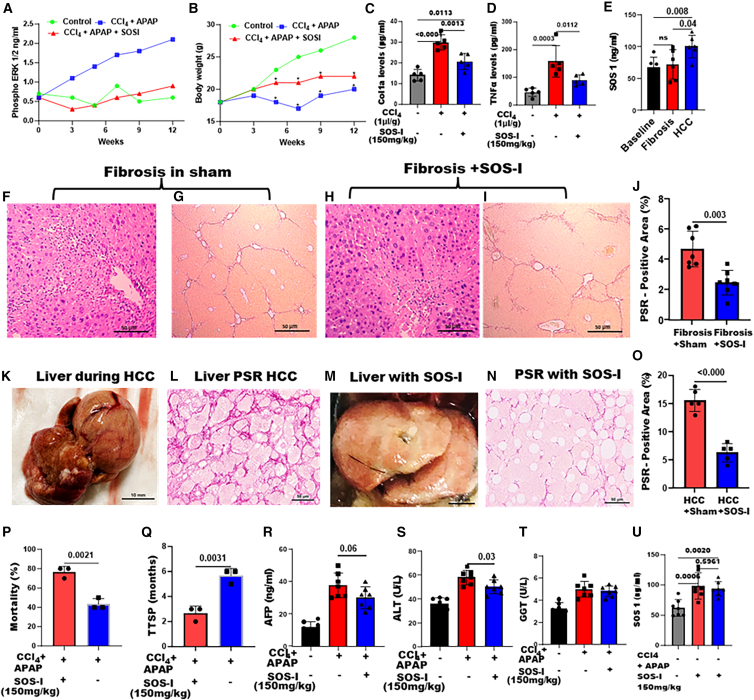
SOS1 inhibition attenuates liver injury, fibrosis, and tumor progression *in vivo* (A and B) Time-course analyses showing phosphorylated ERK1/2 signaling and body weight changes in mice subjected to CCl_4_ + APAP injury with or without SOS1 inhibitor (SOS-I, 150 mg/kg). SOS-I treatment suppressed ERK activation and mitigated weight loss compared to untreated mice. (C and D) Quantification of circulating and tissue biomarkers, including Col1a and TNF-α. (E) Serum SOS1 levels across baseline, fibrosis, and HCC models. (F–J) Representative H&E- and PSR-stained liver sections from control and SOS-I-treated fibrotic mice, with quantitative assessment of collagen deposition shown in (J). (K–N) Gross liver morphology and corresponding histological assessment demonstrating advanced tumor burden and fibrotic remodeling in untreated mice, whereas SOS-I-treated animals display reduced tumor formation and improved tissue integrity. (O) Quantification of fibrosis (PSR-positive area) corresponding to panels (K–N). (P–T) Additional quantitative analyses of disease progression and liver injury markers, including tumor burden and biochemical indicators, showing consistent improvement with SOS1 inhibition. (U) Serum SOS1 levels across experimental groups following intervention. Statistical analyses were performed using one-way ANOVA with Tukey’s post hoc test or two-tailed unpaired Student’s *t* test where appropriate. Data represent mean ± SEM from *n* = 6–8 mice/group (*in vivo* studies). Exact *p* values are shown in the graphs. Scale bars, 50 μm for H&E and PSR histological images (F–I, L, and N) and 10 mm for gross liver morphology images (K and L).

**Figure 10:**
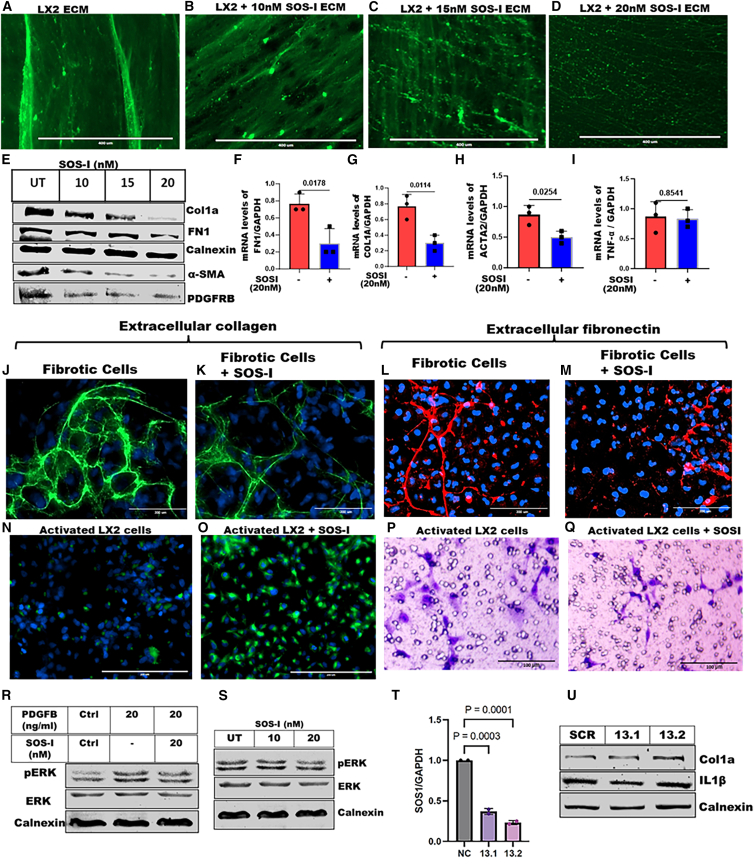
SOS1 inhibition attenuates extracellular matrix deposition, stellate cell activation, and downstream signaling in LX2 hepatic stellate cells (A–D) Decellularized extracellular matrix (ECM) derived from activated LX2 cells treated with increasing concentrations of SOS1 inhibitor (SOS-I) and stained with NHS-Fluorescein, showing a progressive reduction in ECM deposition with SOS1 inhibition. (E) Representative immunoblot of fibrogenic markers (COL1A1, FN1, α-SMA, PDGFRB) with calnexin as loading control, demonstrating reduced protein expression following SOS-I treatment. (F–I) Quantitative analyses of fibrogenic and inflammatory readouts showing modulation of ECM-associated and signaling markers following SOS1 inhibition. (J–M) Immunofluorescence staining of extracellular matrix components, including collagen (green, J and K) and fibronectin (red, L and M), demonstrating reduced matrix deposition in SOS-I-treated cells. Nuclei are counterstained with DAPI (blue). (N and O) BODIPY staining of LX2 cells showing increased lipid droplet accumulation following SOS1 inhibition, consistent with partial reversion of stellate cell activation. (P and Q) Transwell migration assays demonstrating reduced migratory capacity of activated LX2 cells following SOS-I treatment. (R) Immunoblot analysis of pERK and total ERK in LX2 cells stimulated with PDGFB in the presence or absence of SOS-I, with calnexin as loading control, showing attenuation of PDGFB-induced ERK phosphorylation. (S) Immunoblot analysis of pERK and total ERK in LX2 cells treated with increasing concentrations of SOS-I, demonstrating dose-dependent reduction in ERK phosphorylation. (T) qRT-PCR confirmation of siRNA-mediated SOS1 knockdown using two independent siRNAs (13.1 and 13.2), with expression normalized to GAPDH. (U) Immunoblot analysis showing the effect of SOS1 knockdown on COL1A1 and IL-1β protein expression, with calnexin as loading control. Data are presented as mean ± SEM with individual data points shown. Statistical significance is indicated as ∗*p* < 0.05, ∗∗*p* < 0.01. Statistical analyses were performed using one-way ANOVA with Tukey’s post hoc test or two-tailed unpaired Student’s *t* test where appropriate. Data represent mean ± SEM from *n* = 3 independent experiments (LX-2 studies). Exact *p* values are shown in the graphs. Scale bars, 400 μm for ECM imaging in (A–D); 200 μm for extracellular collagen and fibronectin immunofluorescence images (J–M) and BODIPY staining (N and O); and 100 μm for transwell migration assay images (P and Q).

To test causality, we then investigated whether SOS1 was also causally linked to progressive liver disease using a combined carbon tetrachloride (CCl_4_) and acetaminophen (APAP) injury mouse model. Mice were treated with either a SOS1 inhibitor (SOS-I, 150 mg/kg) or vehicle. In fibrotic animals, both sustained ERK1/2 activation and progressive weight loss were observed; both effects were markedly suppressed by SOS-I administration (Figures 9A and 9B). Circulating levels of Col1a and TNF-α were also significantly increased in the control group, which were substantially reduced by SOS-I treatment (Figures 9C and 9D). In addition, serum SOS1 levels were significantly elevated across disease progression from baseline to fibrosis and HCC (Figure 9E). Histological analyses further demonstrated that SOS-I limited fibrotic remodeling. While untreated livers displayed extensive hepatocellular ballooning, bridging fibrosis, and inflammatory infiltrates, SOS-I-treated animals exhibited preserved hepatic architecture with reduced collagen accumulation as confirmed by picrosirius red (PSR) staining and quantification (Figures 9F–9J). In long-term studies, untreated mice developed grossly visible HCC and extensive fibrotic nodularity, whereas SOS-I-treated animals showed preservation of gross liver morphology and markedly reduced sirius red staining of collagen with corresponding quantitative reduction in fibrosis (Figures 9K–9O). Importantly, SOS-I significantly reduced mortality and prolonged time to spontaneous progression (TTSP), highlighting a survival benefit in this preclinical model (Figures 9M and 9N). Circulating biomarkers reflected these protective effects: AFP and alanine transaminase (ALT) levels were decreased in SOS-I-treated mice, while Gamma-glutamyl Transferase (GGT) levels remained unchanged and additional quantitative analyses of tumor burden and biochemical indicators further supported reduced disease severity (Figures 9P–9T). Notably, SOS1 levels remained elevated in untreated animals but were reduced following SOS-I intervention (Figure 9U). Therefore, SOS1 inhibition reduces hepatocellular injury, and delays HCC progression *in vivo*. We next probed how SOS1 affected liver fibrosis and HCC. Despite the complex causes of the disease, a common pathway involves activation of HSCs and ECM accumulation in the liver. Therefore, we assessed whether SOS1 inhibition could reverse the activated HSC phenotype using immortalized human HSCs, LX2 cells, which are widely used for mechanistic studies. We treated LX-2 cells with SOS-I and assessed multiple markers of activation. N-Hydroxysuccinimide (NHS)-fluorescein staining following denuding of cells revealed a marked reduction in ECM deposition following SOS-I exposure (Figures 10A–10D). At the molecular level, immunoblotting and qPCR confirmed downregulation of alpha smooth muscle actin (α-SMA), Col1a, Fn1, and Acta2 transcripts and proteins along with modulation of platelet-derived growth factor receptor beta PDGFRB, a key marker of stellate cell activation (Figures 10E–10I). Confocal immunofluorescence demonstrated decreased accumulation of fibronectin and collagen I under non-permeabilized conditions, consistent with reduced extracellular deposition (Figures 10J–10M). Notably, Boron-Dipyrromethene (BODIPY)-498 staining showed that SOS-I restored lipid droplets in activated LX-2 cells, suggesting a partial reversion toward the quiescent phenotype (Figures 10N and 10M). Functionally, the transwell migration assays demonstrated that SOS-I significantly reduced the migratory capacity of activated LX-2 cells (Figures 10O and 10P). Mechanistically, SOS1 inhibition attenuated downstream RAS–ERK signaling, as demonstrated by reduced ERK phosphorylation following platelet-derived growth factor beta (PDGFB) stimulation (Figure 10R) and in a dose-dependent manner with increasing SOS-I concentrations (Figure 10S). Furthermore, small interfering RNA (siRNA)-mediated knockdown of SOS1 confirmed efficient suppression of SOS1 expression (Figure 10T) and revealed differential downstream effects, with reduction in inflammatory signaling (e.g., IL1β) and more modest effects on structural ECM proteins such as COL1A1 (Figure 10U). Together, these results establish that SOS1 inhibition not only suppresses profibrotic gene expression and ECM production but also restores key features of stellate cell quiescence, supporting its potential as a therapeutic antifibrotic strategy. The results further demonstrated that SOS1 activity is essential for HSC activation, linking clinical observations of elevated SOS1 in cirrhosis and decompensated disease to a mechanistic role in driving ECM deposition. These findings establish SOS1 as both a biomarker of fibrotic progression and a potential therapeutic target to block or revert the activation status of HSCs, a key mechanism that underlies the transition from fibrosis to cirrhosis.

## Discussion

In this translational study spanning human cohorts, preclinical models, and mechanistic studies, we identify SOS1 as a previously unrecognized prognostic marker of liver disease progression. The strength of our findings lies first and foremost in the clinical dimension: in a carefully phenotyped Nigerian cohort, we observed that serum SOS1 increased stepwise from fibrosis to cirrhosis and decompensation, and that its elevation was associated with reduced survival, independent of conventional indices. This observation is important, as existing tools, such as MELD, Child-Pugh, and serum fibrosis markers, primarily reflect liver function or injury but do not fully capture the molecular underpinnings of progression.^14^^,^^15^ Potential confounding by alcohol use, viral hepatitis status, and metabolic comorbidities was addressed through prespecified covariate adjustment guided by DAGs and further mitigated using inverse probability weighting; however, residual confounding cannot be excluded. By directly linking SOS1 to both disease stage and outcome, our study introduces a novel biomarker that has potential to refine risk stratification in routine clinical care. Although the primary cohort was geographically localized, consistency across TCGA validation and experimental models supports broader biological relevance. Unlike many prior biomarker studies limited to cirrhosis or HCC, our cohort encompassed the full spectrum of chronic liver disease, from early fibrosis to compensated cirrhosis and overt decompensation.^16^ This design allowed us to make stage-specific comparisons and to demonstrate that SOS1 expression remains uniformly low in fibrosis but rises sharply at the point of decompensation. This pattern suggests that SOS1 induction marks the transition to clinically significant disease. Beyond stage-specific associations, the longitudinal extension in 117 patients with decompensated cirrhosis provided a unique vantage point to interrogate the dynamics of SOS1 as a determinant of survival. Unlike conventional metrics such as the MELD score or AFP, which capture static or injury-related signals, SOS1 trajectories embodied the molecular tempo of disease. Patients exhibiting steep SOS1 rises across 24–48 months faced sharply elevated mortality by 60 months, whereas stable profiles conferred relative protection. Receiver operating characteristic and calibration analyses underscored that this signal was both discriminatory and well aligned with observed outcomes, while trajectory clustering revealed distinct biological phenotypes hidden to traditional scores. These data not only extend SOS1 beyond a cross-sectional marker of decompensation but also introduce it as a dynamic molecular barometer of progression, reframing how risk can be captured and predicted in advanced liver disease.^17^^,^^18^

To ensure that our findings were not cohort-specific, we extended the analysis to the publicly available TCGA-LIHC dataset. Here, SOS1 expression was consistently elevated in tumor tissue compared with adjacent normal controls, demonstrated superior diagnostic accuracy compared to AFP and other matrix-related genes, and remained an independent predictor of survival after adjustment for age and sex. Correlation analyses confirmed that SOS1 clustered most strongly with FN1 and COL1A1, further underscoring its role in fibrogenic remodeling.^19^ The convergence of results from serum biomarker discovery in our clinical study and independent validation in TCGA lends credibility to SOS1 as a robust biomarker across distinct patient populations and biological matrices. The external validation also addresses a common limitation of early biomarker studies, which often lack replication in large independent datasets.^20^^,^^21^

The *in vivo* experiments extend these observations to a therapeutic context. In a combined CCl_4_ and acetaminophen injury model designed to capture both toxic and inflammatory drivers of chronic liver disease, SOS1 inhibition attenuated fibrosis, reduced hepatocellular injury, preserved liver architecture, delayed hepatocarcinogenesis, and improved survival. These results are particularly notable given the difficulty of demonstrating survival benefits in preclinical liver disease models.^22^^,^^23^^,^^24^ Furthermore, SOS1 inhibition was well tolerated in our murine system, consistent with early-phase oncology trials of SOS1 inhibitors showing favorable safety profiles.^25^ This pharmacological precedent enhances the translational relevance of our findings, as it suggests that SOS1-targeted therapies could be repurposed for liver disease without the need to develop entirely new drug classes. Notably, SOS2, a close family member, was not detectable in circulation and did not exhibit compensatory upregulation following SOS1 perturbation, suggesting limited involvement of SOS2 in the circulating and functional dynamics observed in this study. From a mechanistic standpoint, our work provides the first evidence that SOS1 activity regulates stellate cell activation and ECM accumulation. *In vitro*, pharmacological inhibition of SOS1 suppressed α-SMA expression, blunted fibrogenic gene transcription, and reduced deposition of collagen and fibronectin, even under strong profibrotic stimulation by TGFβ.^26^^,^^27^ These findings suggest that SOS1 functions as an essential integrator of upstream fibrogenic cues. SOS1 is known to be compartmentalized in the cytoplasm under basal conditions, but translocates to the plasma membrane during liver injury, where it integrates receptor tyrosine kinase signals into downstream ERK and AKT pathways.^28^ Consistent with the role of SOS1 as an upstream regulator of RAS-ERK signaling, modulation of SOS1 was associated with corresponding changes in ERK phosphorylation, providing functional evidence of pathway engagement despite limitations in direct protein detection. This spatial regulation provides a mechanistic explanation for our observation that SOS1 induction coincides with disease progression that once mobilized to the plasma membrane, SOS1 amplifies profibrotic signaling and reinforces stellate cell survival and proliferation.

In conclusion, this work introduces SOS1 as a novel biomarker and therapeutic target in progressive liver disease. By integrating human clinical data, *in vitro* assays, and *in vivo* therapeutic modeling, we define SOS1 as a key regulator linking stellate cell activation to fibrosis progression and adverse clinical outcomes. The dual role of SOS1 as both a prognostic biomarker and a druggable node in fibrogenic signaling sets it apart from conventional biomarkers and opens new avenues for risk stratification and therapy. Our findings establish SOS1 as both a clinically relevant biomarker and a mechanistically actionable target in chronic liver disease. Clinically, serum SOS1 provides stage-specific prognostic information, identifying patients at high risk of decompensation and death beyond conventional indices such as MELD, Child-Pugh, or AFP. Longitudinal profiling in an extended cohort demonstrated that steep SOS1 trajectories predicted mortality with greater precision than static biochemical scores, while clustering analyses revealed biologically distinct subgroups with divergent outcomes. External validation in TCGA-LIHC confirmed that SOS1 outperformed AFP and other ECM-associated genes in diagnostic and prognostic accuracy. Mechanistically, SOS1 inhibition *in vivo* mitigated fibrosis, reduced hepatocellular injury, preserved lobular architecture, delayed hepatocarcinogenesis, and improved survival; *in vitro*, inhibition suppressed stellate cell activation and ECM synthesis. By integrating human cohorts, preclinical models, and mechanistic assays, this study defines SOS1 as a dynamic molecular barometer and druggable regulator of progression, reframing risk stratification and therapeutic intervention in liver disease.

### Limitations of the study

This study integrates a large, well-characterized clinical cohort with complementary preclinical and mechanistic models, providing convergent evidence that SOS1 drives both disease progression and therapeutic response. While the analyses span multiple time points and disease stages, further validation in geographically diverse populations will help define broader clinical applicability. The modeling framework focused on discrimination, calibration, and dynamic prediction, which can be extended prospectively to evaluate decision thresholds in trial settings. Mechanistic interrogation combined pharmacologic inhibition and translational *in vitro* assays, but additional pathway-mapping studies may further delineate SOS1’s signaling context within RAS-ERK and RAS-AKT cascades. Overall, the integration of human, *in vivo*, and cellular data provides a comprehensive and coherent framework that supports the translational advancement of SOS1 as both a biomarker and therapeutic target in chronic liver disease.

## Resource availability

### Lead contact

Requests for further information and resources should be directed to and will be fulfilled by the lead contact, Peter U. Amadi (pamadi@ualberta.ca).

### Materials availability

This study did not generate new unique reagents.

### Data and code availability

• Publicly available TCGA-LIHC datasets used in this study were accessed through The Cancer Genome Atlas portal.
• This paper does not report original code.
• Any additional information required to reanalyze the data reported in this paper is available from the lead contact upon request.

## Acknowledgments

We are grateful to the clinicians, and research coordinators who supported patient recruitment, clinical follow-up, and biospecimen collection across the participating centers. We acknowledge the invaluable contributions of the hepatology and gastroenterology staff of Imo State University and her collaborating hospitals, whose expertise ensured rigorous clinical phenotyping and patient care throughout the study. We also thank the technical staff of the Departments of Pathology and Laboratory Medicine for their assistance with histological staining, biomarker assays, and imaging support. The dedicated efforts of our animal facility personnel are recognized for ensuring high standards in the conduct of *in vivo* experiments. We are indebted to the members of the Institutional Clinical Trial/Research Oversight Committee for their oversight and guidance, and to our colleagues in the Faculty of Biomedical Sciences for providing essential laboratory infrastructure. Finally, we extend our gratitude to all patients and their families, whose participation made this research possible.

## Author contributions

Conceptualization, P.U.A.; methodology, P.U.A., C.W.A., G.S.G., J.O.O., P.C.O., S.J.J., R.M.P., M.L., C.N.E., M.U.E., and J.A.A.; investigation, P.U.A., H.-m.G., E.N.A., and D.-w.Z.; writing – original draft, P.U.A.; writing – review and editing, P.U.A., D.-w.Z., A.O’B., B.d.C., and R.W.; funding acquisition, P.U.A., J.O.O., and D.-w.Z.; resources, P.U.A., J.O.O., D.-w.Z., E.N.A., C.N.E., and M.U.E.; supervision, P.U.A., J.O.O., J.A.A., D.-w.Z., E.N.A., and P.C.O.

## Declaration of interests

The authors declare no competing interests.

## STAR★Methods

### Key resources table

**Table:** 

REAGENT or RESOURCE	SOURCE	IDENTIFIER
**Antibodies**		

Collagen Type I Polyclonal antibody	ProteinTech	Catalog # 14695-1-AP; RRID:AB_2082037
Collagen Type I Polyclonal antibody	ProteinTech	Catalog # 66761-1-Ig; RRID:AB_2882107
Anti-Calnexin antibody - ER Marker	Abcam	Catalog # ab22595; RRID:AB_2069006
Calnexin Polyclonal antibody	ProteinTech	Catalog # 10427-2-AP; RRID:AB_2069033
Fisher BioReagents™ EZ-Run™ Prestained Rec Protein Ladder	Thermofisher	Catalog # BP3603500
BLUeye Prestained Protein Ladder	Sigma	Catalog # 94964
Fibronectin Polyclonal antibody	ProteinTech	Catalog # 15613-1-AP; RRID:AB_2105691
Alpha smooth muscle actin Polyclonal antibody	ProteinTech	Catalog # 14395-1-AP; RRID:AB_2223009
Phospho-p44/42 MAPK (Erk1/2) (Thr202/Tyr204) Antibody	Cell Signaling	Catalog # 9101; RRID:AB_331646
p44/42 MAPK (Erk1/2) (137F5) Rabbit Monoclonal Antibody	Cell Signaling	Catalog # 4695; RRID:AB_390779
IL-1 beta Polyclonal antibody	ProteinTech	Catalog # 16806-1-AP; RRID:AB_10646432
Goat anti-Rabbit IgG (H + L) Cross-Adsorbed Secondary Antibody, Alexa Fluor™ 488	Thermofisher	Catalog # A-11008
Goat anti-Rabbit IgG (H + L) Highly Cross-Adsorbed Secondary Antibody, Alexa Fluor™ 546	Thermofisher	Catalog # A-11035

**Biological samples**		

Whole Blood and Plasma	Participants in the cohort	ID: BCH/203/1-949
Mice plasma	Experimental animals in this study	ID: BCH/11-03/1-79
Mice liver	Experimental animals in this study	ID: BCH/11-03/L1-79

**Chemicals, peptides, and recombinant proteins**		

Fetal Bovine Serum	Sigma	Catalog # 12133C
Penicillin-Streptomycin	Thermofisher	Catalog # 15140-122
Human TGF-beta 1 Recombinant Protein	Thermofisher	Catalog # 100-21-10UG
BI-3406	Boehringer/MedChemExpress	Catalog # HY-125817
ASP2453	MedChemExpress	Catalog # HY-132966
NHS-Fluorescein	ThermoFisher	Catalog # 46409
VECTASHIELD Antifade Mounting Medium with DAPI	Vector Laboratories	Catalog # H-1200-10
Carbon-13C tetrachloride	Sigma	Catalog # 488488-500 MG
Acetaminophen	Sigma	Catalog # A5000
Human PDGF-BB Recombinant Protein	ThermoFisher	Catalog # 100-14B-10UG

**Critical commercial assays**		

Alpha-Fetoprotein Kit	R&D Systems	Catalog #: DAFP00
Fibronectin DuoSet Kit	R&D Systems	Catalog #: DY1918-05
Hyaluronan Kit	R&D Systems	Catalog #: DHYAL0
Tumor Necrosis Factor DuoSet Kit	R&D Systems	Catalog #: DY210
Interleukin 1 Beta DuoSet Kit	R&D Systems	Catalog #: DY201
Interferon Gamma Kit	R&D Systems	Catalog #: DIF50C
Matrix metalloproteinase 2 kit	Sigma	Catalog # RAB0365
Matrix metalloproteinase 9 kit	Abcam	Catalog # ab246539
Tissue Inhibitor of Metalloproteinases 1 Kit	Abcam	Catalog # ab187394
Human Son of Sevenless Homolog 1 Kit	Abbkine	Catalog #KTE60555
Human Son of Sevenless Homolog 2 Kit	Abbkine	Catalog #KTE60554
Mouse alpha Fetoprotein ELISA Kit	Abcam	Catalog # ab210969
Mouse Pro-Collagen I alpha 1 ELISA Kit	Abcam	Catalog # ab210579
Mouse TNF alpha ELISA Kit	Abcam	Catalog # ab208348

**Experimental models: Cell lines**		

LX-2 Human Hepatic Stellate Cell Line	Sigma	Catalog # SCC064; RRID:CVCL_5792

**Experimental models: Organisms/strains**		

Male C57BL/6J mice	Jackson Lab	CCID: 9137109-9137183

### Experimental model and study participant details

#### Human participants

This study included 556 adult patients with clinically and histologically characterized chronic liver disease prospectively recruited from tertiary liver centers in Imo and Rivers States, Nigeria, between May 2017 and December 2019. Participants were stratified into fibrosis, cirrhosis, and hepatocellular carcinoma (HCC) groups according to histologic staging and clinical criteria. Eligible participants were 18–75 years old and provided written informed consent prior to enrollment. The study protocol was approved by the institutional research ethics board at Imo State University (reference number IMSU/ETS/BCH/203) and conducted in accordance with the Declaration of Helsinki and Good Clinical Practice guidelines. Sex was incorporated into multivariable and subgroup analyses, including interaction-term analyses evaluating effect consistency across sex-defined subgroups. Ethnicity-specific analyses were not performed and represent a limitation regarding broader generalizability.

#### Experimental animals

Male C57BL/6J mice (8–10 weeks old; 20–25 g; Jackson Laboratory) were used for *in vivo* fibrosis and therapeutic studies. Animals were housed under specific pathogen-free conditions in individually ventilated cages under controlled temperature and 12-h light/dark cycling with *ad libitum* access to chow and water. All animal procedures complied with the Guide for the Care and Use of Laboratory Animals and were approved by the Institutional Animal Care and Use Committee (protocol ID: IMSU/ETS/BCH/11-03). Animals were randomized into experimental groups, and investigators were blinded during outcome assessment.

#### Cell lines

Human hepatic stellate LX-2 cells (Millipore SCC064; male donor-derived) and HepG2 hepatoma cells were used for mechanistic *in vitro* studies. Cells were cultured under standard conditions at 37 °C with 5% CO_2_ using DMEM supplemented with fetal bovine serum and penicillin-streptomycin as described in the STAR Methods section. SOS1 inhibition, siRNA-mediated knockdown, migration assays, extracellular matrix imaging, and pathway activation experiments were performed in LX-2 cells.

### Method details

#### Ethics statement

The study protocol was approved by the institutional research ethics board at Imo State University, reference number IMSU/ETS/BCH/203. All participants provided written informed consent prior to enrollment, and the study was conducted in accordance with the Declaration of Helsinki and Good Clinical Practice guidelines.

#### Clinical cohort and study design

A total of 556 patients with histologically and clinically characterized liver disease were prospectively recruited from tertiary liver centers in Imo and Rivers States, Nigeria, between May 2017 and December 2019. Participants were stratified into four primary disease categories based on histologic staging and clinical criteria: F0-F1 (*n* = 146) representing mild fibrosis, F2-F3 (*n* = 130) representing moderate fibrosis, F4 (*n* = 152; decompensated *n* = 34 (22.4%)) denoting cirrhosis, and HCC (*n* = 128) representing hepatocellular carcinoma. Patients with decompensated cirrhosis were included within the F4 category and further characterized according to ascites, encephalopathy, or variceal bleeding at follow-up. Eligible participants were aged 18–75 years and free of coexistent inflammatory or neoplastic conditions. Exclusion criteria included prior liver transplantation, active infection, antiviral or antifibrotic therapy, or incomplete longitudinal data. All patients underwent standardized baseline evaluation followed by serial follow-up data collection at 0, 12, 24, and 36 months. At each timepoint, clinical assessment, and biochemical profiling, and adverse events including decompensation, HCC onset, and mortality were recorded. Physical examination included evaluation for hepatomegaly, splenomegaly, ascites, and encephalopathy (graded using West Haven criteria). Endoscopy performed in cirrhotic and decompensated patients defined varices whereas imaging (ultrasound) defined the liver morphology, splenomegaly, ascites, and to exclude HCC at baseline. At each visit, standardized case report forms recorded interim decompensation events, hospitalizations, and survival status. Survival was defined as time from enrollment to death or liver transplantation, with censoring at last follow-up. Time-to-spontaneous progression (TTSP) was defined as the interval from baseline staging (fibrosis, cirrhosis) to the first documented decompensating event. A nested sub-cohort of individuals with complete serial biomarker data (*n* ≈ 420) contributed to landmark and slope-based analyses, while the full dataset informed cross-sectional, spline, and calibration models.

#### Extended cohort for mortality prediction

In addition to the 556 participants described above, an independent cohort of 117 patients with decompensated cirrhosis was assembled to assess the prognostic role of serum SOS1 in predicting mortality. Patients were enrolled between 2018 and 2023 and followed longitudinally with blood sampling at baseline, 24 months, and 48 months, with survival status determined at 60 months. Serum SOS1, AFP, and MELD were quantified at each timepoint. Biomarker slopes (Δ0-24m, Δ24-48m) were calculated to capture dynamic changes. Statistical analyses were performed to evaluate discrimination, calibration, and incremental utility of SOS1. Logistic regression models were fit at each landmark and slope window, with nested models constructed for MELD alone, MELD+SOS1, MELD+AFP, and MELD+SOS1+AFP. Model performance was quantified by the area under the receiver operating characteristic curve (AUC), with bootstrapped 95% confidence intervals. Incremental improvement was assessed using net reclassification index (NRI) and integrated discrimination index (IDI). Calibration was evaluated using calibration slopes, intercepts, and calibration curves based on deciles of predicted risk. Trajectory clustering was performed using k-means on standardized SOS1 trajectories (0, 24, and 48 months) to identify patient subgroups, with mortality rates compared between clusters.

#### Laboratory measurements

Peripheral venous blood was collected after overnight fasting and processed within 2 h. Routine hematology and chemistry analyses were performed using Sysmex XN-1000 and Roche Cobas 8000 systems. Coagulation parameters (PT/INR) and fasting insulin were measured by standard immunoassay. The FIB-4 and MELD indices were calculated as follows:

**FIB-4:** (Age × AST [U/L]) ÷ (Platelets [10^9^/L] × √ALT [U/L])
**MELD:** 3.78 × ln(bilirubin [mg/dl]) + 11.2 × ln(INR) + 9.57 × ln(creatinine [mg/dl]) + 6.43.

#### Serum biomarker assays

Serum was separated by centrifugation (1,500 g, 15 min, 4 °C) and stored at −80 °C in aliquots to avoid repeated freeze-thaw cycles. ELISAs were performed in duplicate according to manufacturer’s protocols for AFP, fibronectin (FN1), hyaluronic acid, TNFα, IL-1β, IFNγ, MMP2, MMP9, and TIMP1 (kits from R&D Systems, and Abcam; catalog numbers listed in key resources table). Inter-assay and intra-assay coefficients of variation were <10%. ALT, AST, GGT, and INR were measured in certified hospital laboratories. Serum SOS1 was quantified using a sandwich ELISA kit (Abbkine, Human Son of Sevenless Homolog 1 [SOS1] ELISA Kit, Cat. #KTE60555) according to the manufacturer’s protocol (with full details of assay validation, in the supplementary file).

#### *In vitro* and *in vivo* experiments

##### LX-2 cell culture and SOS1 inhibition

Human hepatic stellate cells (LX-2; Millipore SCC064; derived from a male donor) were cultured in DMEM supplemented with 2% fetal bovine serum (Sigma 12133C) and 1% penicillin-streptomycin (Gibco 15140-122) at 37 °C and 5% CO_2_. The cells were activated 24h after setting, with 2.5 ng/ml TGFβ. SOS1 inhibitor (SOS-I; [Bl-3406, Boehringer) was dissolved in DMSO and applied at 10–20 nM for 24–72 h. A second SOS-I, HY-132966 (MedChem Express), was used for confirmation. For ECM extraction confluent cells were decellularized with 0.5% Triton X-100/20 mM NH_4_OH for 5 min, rinsed, and stained with NHS-Fluorescein (ThermoFisher 46409).^2^ For pathway activation studies, cells were stimulated with PDGFB (20 ng/mL), and ERK phosphorylation (pERK) was assessed 30 min post-stimulation. Proteins were extracted in RIPA buffer supplemented with protease/phosphatase inhibitors. Western blotting was performed using antibodies against COL1A1 (Proteintech 14695-1-AP/66761-1-Ig)), FN1 (Proteintech 15613-1-AP), α-SMA (Proteintech 14395-1-AP), and Calnexin (Abcam ab22595; ProteinTech 10427-2-AP). RNA was extracted (Qiagen RNeasy), reverse transcribed (Applied Biosystems), and quantified by SYBR-Green qPCR with primers targeting FN1, COL1A1, ACTA2, and TNF. For siRNA-mediated knockdown experiments, LX-2 cells were transfected with two independent SOS1-targeting siRNAs (13.1 and 13.2) or scrambled control (SCR) using Lipofectamine RNAiMAX (ThermoFisher) according to the manufacturer’s instructions and analyzed 48–72 h post-transfection. siRNA sequences are provided in Table S1. The primers sequence is included in Table S1.

##### Transwell migration assay

Transwell migration was assayed using 8 μm inserts (12-well format). Serum-starved LX2 cells (5 × 10^4^-1×10^5^/insert) were seeded into the upper chamber in serum-free DMEM ± SOS-I (10–20 nM), with a chemotactic gradient of TGF-β1 (2.5 ng/mL) in 0.5–1% FBS DMEM in the lower chamber; matching vehicle or SOS-I was included in both chambers. After 24 h at 37 °C, non-migrated cells were removed from the upper surface, membranes were fixed (70% ethanol) and stained with crystal violet, and migrated cells on the underside were quantified (≥4 fields/insert) as cells/mm^2^. Data represent mean ± SEM from ≥3 independent experiments.

##### Non-permeabilized confocal microscopy

Extracellular fibronectin and collagen I were visualized under non-permeabilized conditions. LX-2 cells (1×10^5^/well) were activated with TGF-β1 (2.5 ng/mL, 24 h, 6 days) and treated with SOS-I (10–20 nM) or vehicle during the final 72 h. Cells were fixed in 4% paraformaldehyde (PFA) for 30 min, blocked (10% goat serum), and incubated overnight (4 °C) with rabbit anti-FN1 and rabbit anti-COL1A1 primaries, followed by Alexa Fluor 568 anti-rabbit and Alexa Fluor 488 anti-mouse secondaries (1 h, room temperature). No detergent was used at any step. Coverslips were mounted with VECTASHIELD Antifade Mounting Medium with DAPI and imaged by confocal microscopy (sequential acquisition; pinhole 1 AU). ECM signal was quantified as area fraction and integrated density (Fiji) and normalized to vehicle.

#### *In vivo* fibrosis and SOS1 inhibitor treatment

##### Animals

Male C57BL/6J mice (8–10 weeks, 20–25 g; Jackson Laboratory) were housed under specific pathogen-free conditions in individually ventilated cages with 12-h light/dark cycles, 22 ± 1 °C temperature, and *ad libitum* access to chow and water. All procedures complied with the Guide for the Care and Use of Laboratory Animals and were approved by the Institutional Animal Care and Use Committee (protocol ID: IMSU/ETS/BCH/11-03). Animals were randomly allocated to experimental groups, and investigators were blinded to group assignment during outcome assessment.

##### Fibrosis induction

Liver fibrosis was induced by combined chronic administration of carbon tetrachloride (CCl_4_) and acetaminophen (APAP) to mimic mixed toxic and inflammatory injury. CCl_4_ (1 μL/g body weight, diluted 1:3 in corn oil) was administered intraperitoneally twice weekly for 8 weeks. Beginning in week 3, APAP (150 mg/kg in sterile saline, freshly prepared) was given by oral gavage once weekly to enhance hepatocellular stress. Control mice received vehicle injections (corn oil + saline) on the same schedule.

##### SOS1 inhibitor treatment

For therapeutic intervention, mice received oral SOS1 inhibitor (MRTX0902; 150 mg/kg/day in 0.5% methylcellulose) or vehicle during the final 4 weeks (150 mg/kg/day) via gavage for the final 4 weeks of the 8-week CCl_4_ + APAP protocol. SOS-I was prepared fresh daily in 0.5% methylcellulose. Vehicle-treated fibrotic mice received 0.5% methylcellulose alone. Dose selection was based on published pharmacokinetic data and pilot tolerability studies.^10^

##### Sample size and monitoring

A minimum of 6–8 mice per group was used to ensure statistical power to detect a ≥25% reduction in collagen content with α = 0.05 and 80% power. Animals were randomized by computer-generated sequence; outcome assessors for histology and biochemistry were blinded. Mice were monitored daily for activity, grooming, body weight, and signs of distress. Body weight was recorded weekly. Humane endpoints (≥20% body weight loss, severe lethargy, inability to feed) triggered euthanasia by CO_2_ inhalation.

##### Serum biochemistry

At euthanasia, blood was collected by cardiac puncture under isoflurane anesthesia, centrifuged at 2,000g for 20 min, and stored at −80 °C. Serum ALT, AST, GGT, bilirubin, and albumin were quantified using automated analyzers (Roche Cobas 8000). Serum α-fetoprotein (AFP), Col1a1, and TNFα were measured by ELISA (Abcam, ab210969, ab210579, ab208348, respectively).

##### Histology and fibrosis assessment

Livers were excised, weighed, and divided for histology, biochemical assays, and RNA/protein extraction. Sections from the left and median lobes were fixed in 10% neutral-buffered formalin, paraffin-embedded, and cut at 5 μm. Hematoxylin-eosin (H&E) staining assessed architecture and necroinflammation. Picrosirius red (PSR) staining quantified collagen deposition, imaged under a light microscope. Fibrosis was quantified as percentage-stained area using ImageJ (NIH) on ≥10 random high-power fields per lobe.

##### Molecular analyses

Snap-frozen liver tissue was homogenized in TRIzol (Invitrogen), and RNA was extracted per manufacturer’s protocol. Reverse transcription was performed with the SuperScript IV kit (Invitrogen), followed by qPCR using SYBR Green (Applied Biosystems). Primers targeted Fn1, Col1a1, Acta2, Mmp2, Mmp9, Timp1, and Gapdh. Protein lysates were prepared in RIPA buffer with protease/phosphatase inhibitors and immunoblotted for SOS1, *p*-ERK1/2, α-SMA, Col1a1, FN1, and GAPDH.

##### Survival and tumor monitoring

Mice were followed for up to 20 weeks post-CCl_4_ initiation. Survival was recorded and analyzed by Kaplan-Meier methods. Gross liver morphology was documented, and nodules >2 mm were classified as hepatocellular carcinoma (HCC) by histopathological confirmation. Time-to-spontaneous progression (TTSP) was defined as the interval from fibrosis induction to development of advanced fibrosis or HCC.

### Quantification and statistical analysis

Statistical and Longitudinal Modeling Framework Longitudinal changes at 0, 12, 24, and 36 months were analyzed by repeated-measures ANOVA and mixed-effects models with random subject intercepts. Causal inference was implemented using directed acyclic graphs to specify minimal adjustment sets, followed by inverse-probability-of-treatment weighting (IPTW) to balance covariates across biomarker strata. Covariate balance was verified using standardized mean differences and effective sample-size metrics. Continuous risk relationships were modeled via Cox proportional-hazards regression with restricted cubic splines (four knots) at baseline, 12 m, and 24 m, adjusted for age, sex, BMI, diabetes, MELD, and etiology. Prognostic discrimination was assessed by time-dependent AUC(t) and optimism-corrected C-index from 1000 bootstraps. Model calibration was evaluated at 12, 24, and 36 months using logistic recalibration and calibration belts. Incremental predictive value was assessed via category-free NRI and IDI against MELD ± AFP or FIB-4 models. Net clinical benefit was estimated by decision-curve analysis across probability thresholds of 0–40%. Individual biomarker slopes (12–36 m) were calculated by linear regression and standardized to 1-SD increments before entry into Cox models. Fine-Gray subdistribution models quantified cumulative incidence of HCC and death across biomarker tertiles and landmarks (0, 12, 24 m).

#### Calibration, subgroup, and enrichment analyses

Calibration belts visualized model agreement with observed outcomes at 12, 24, and 36 months. Two-dimensional risk heatmaps integrated standardized SOS1 and AFP z-scores to estimate joint risk surfaces. Subgroup analyses by sex, diabetes status, BMI (<30 vs. ≥ 30 kg/m^2^), and etiology tested effect consistency using interaction terms. For translational modeling, enrichment analyses estimated event-rate concentration and sample-size efficiency under assumed 20% and 30% relative-risk reductions. Percentile thresholds of SOS1, AFP, and FIB-4 defined enrichment strata, from which required trial N, fold-enrichment, and screening penalties were computed using binomial variance approximations and Monte Carlo simulations. All analyses were performed in R (v4.3.2) and GraphPad Prism (v9.4). Normality was tested by Shapiro-Wilk, and all *p* values were two-sided with α = 0.05 after FDR correction.

#### Data analyses and data visualization

All analyses were performed in R (v4.3.2, R Foundation for Statistical Computing) and GraphPad Prism (v9.4, GraphPad Software). Figures were generated using the ggplot2, survival, and time-ROC packages. Continuous variables are expressed as mean ± SEM for normally distributed data or median (IQR) for non-normal distributions. Normality was assessed by Shapiro-Wilk testing. Between-group comparisons used two-tailed *t* test or one-way ANOVA with Tukey post-hoc correction for normally distributed data and Mann-Whitney U or Kruskal-Wallis tests with Dunn’s correction for non-parametric data. Repeated measures across 0–36 months were modeled with linear mixed-effects models including random subject intercepts. Correlations between continuous biomarkers were evaluated by Pearson or Spearman methods, with Benjamini-Hochberg FDR adjustment for multiple testing. Survival and time-to-progression were analyzed by Kaplan-Meier and log rank tests, with multivariable Cox proportional-hazards models adjusted for prespecified covariates. Proportional-hazards assumptions were verified using Schoenfeld residuals. Diagnostic and prognostic accuracy were quantified by ROC analysis with area under the ROC curve (AUROC) and 95% CIs; between-marker differences were tested using DeLong’s method. Given the available sample size (*n* = 556), no formal *a priori* power calculation was performed, and all analyses are considered exploratory yet adequately powered for observed effect sizes.
