# Supplementary material for: Serum SOS1 as a prognostic biomarker and therapeutic target for progressive liver disease
Source: iScience. 2026 Jun 17;29(7):116430. doi: 10.1016/j.isci.2026.116430 (PMC13293661; doi:10.1016/j.isci.2026.116430)
Supplement: Document S1. Figures S1–S8, Tables S1–S5, and Methods S1–S3 [file mmc1.pdf]

**iScience, Volume *29***

## Supplemental information

# Serum SOS1 as a prognostic biomarker and therapeutic target for progressive liver disease

**Peter U. Amadi, Govind S. Gill, Chiamaka W. Amadi, Justice O. Osuoha, Prince C. Odika, Raj M. Patel, Suha J. Jarad, Hong-mei Gu, Moattar Latif, Joy A. Amadi, Celestine N. Ekweogu, Melford U. Elendu, Emmanuel N. Agomuo, Alastair O'Brien, Barbora de Courten, Ralf Weiskirchen, and Da-wei Zhang**

**Supplementary material**

**Figure S1: Longitudinal dynamics of extracellular matrix biomarkers across fibrosis stages.**

**Figure S2: Temporal trajectories of metalloproteinase regulators across fibrosis progression.**

**Figure S3: Distribution and temporal stability of metalloproteinase regulators across disease stages.**

**Figure S4: Calibration, joint-risk structure, and subgroup effects for SOS1-based prognostic modeling.**

**Figure S5: Trial enrichment and sample-size optimization using SOS1-based risk thresholds.**

**Figure S6: Representative original uncropped Western blots (to Figure 10e)**

**Figure S7: Representative original uncropped Western blots (to Figure 10e,r,s,t)**

**Figure S8: Quantitative analysis of gene expression following SOS1 knockdown using two independent siRNAs (13.1 and 13.2).**

**Table S1: List of Primers**

**Table S2: ROC Summary table**

**Table S3: Landmark risk sets and prediction horizon**

**Table S4: Decision-curve analysis (net benefit) for 48→60-month mortality among 48-month survivors**

**Table S5: Landmark and slope performance for 48→60-month mortality in 48-month survivors**

**Method S1: Serum SOS1 ELISA and assay validation**

**Method S2: Performance characteristics**

**Method S3: Matrix and handling controls**

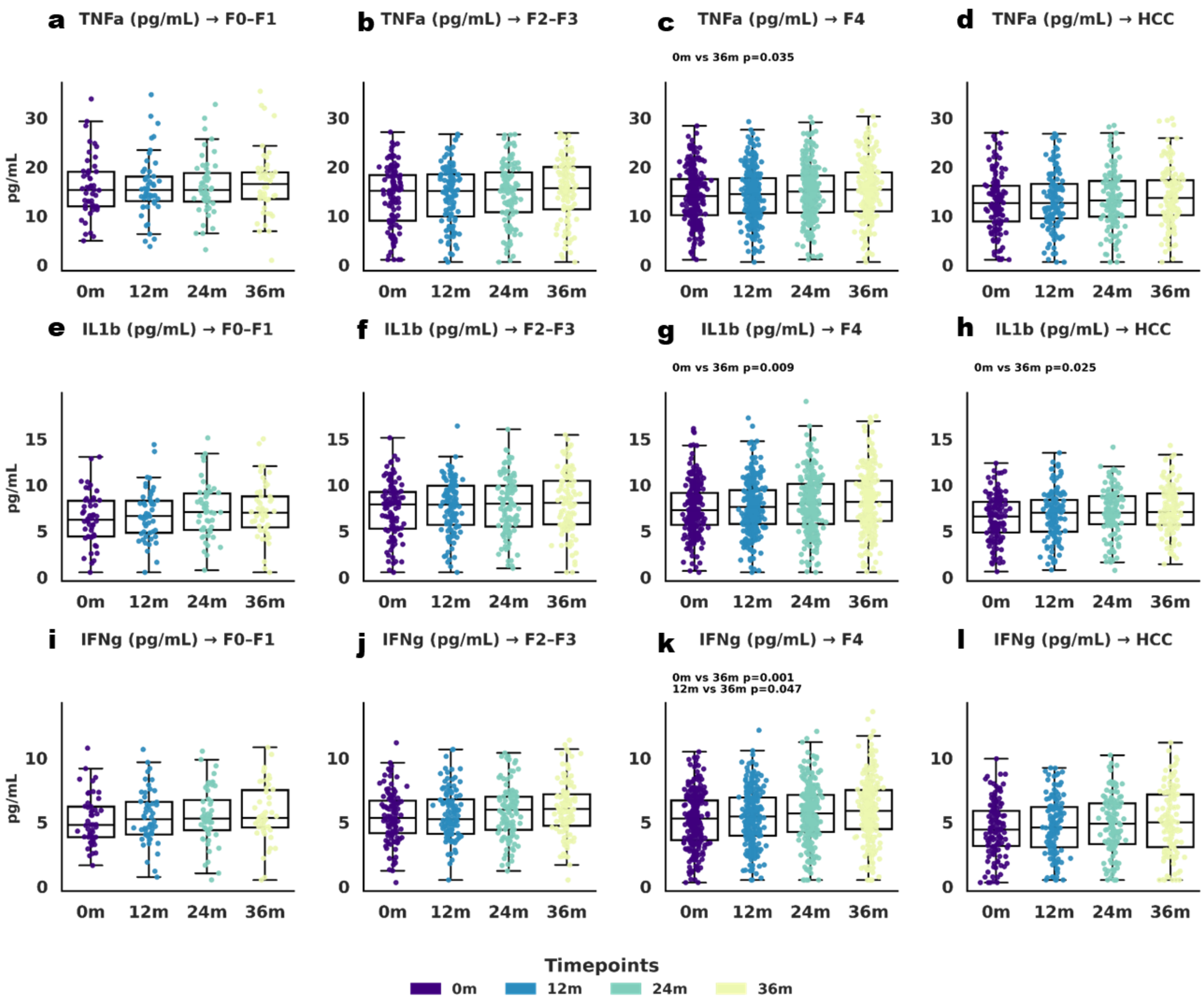


**Figure S1:** Longitudinal dynamics of extracellular matrix biomarkers across fibrosis stages. Box-and-whisker plots show serial serum levels of COL1A1 (a-d), fibronectin (FN1; e-h), and hyaluronic acid (i-l) measured at baseline (0 m), 12 m, 24 m, and 36 m in participants stratified by histological or clinical stage (F0-F1, F2-F3, F4, and HCC). Each dot represents an individual value, boxes indicate the interquartile range with median lines, and whiskers extend to 1.5× IQR. Timepoints are color-coded (0 m, dark violet; 12 m, cyan; 24 m, light green; 36 m, yellow). P values reflect within-stage one-way ANOVA with Tukey-adjusted post-hoc comparisons across timepoints. COL1A1 and FN1 concentrations increased progressively with advancing disease and time, whereas hyaluronic acid exhibited modest yet significant late-stage elevations. All data are presented on native concentration scales without transformation, and comparisons were performed independently within each fibrosis stratum.

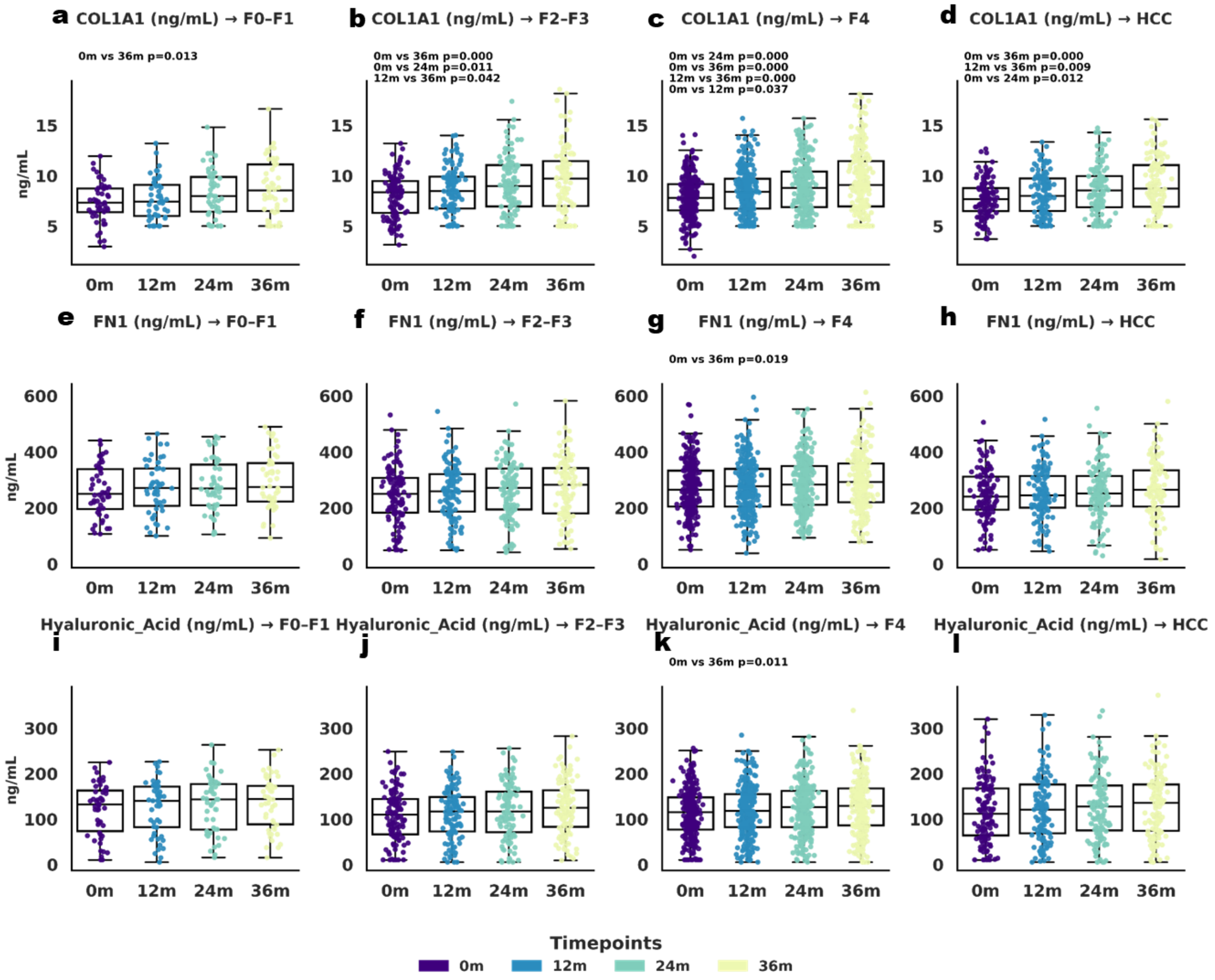


**Figure S2:** Temporal trajectories of metalloproteinase regulators across fibrosis progression. Longitudinal serum concentrations of TIMP2 (a-d), MMP2 (e-h), and MMP9 (i-l) were quantified at baseline (0 m), 12 m, 24 m, and 36 m in participants stratified by histological or clinical stage (F0-F1, F2-F3, F4, and HCC). Each dot represents an individual participant value, boxes indicate the interquartile range with the median, and whiskers extend to 1.5× IQR. Timepoints are color-coded (0 m, dark violet; 12 m, cyan; 24 m, pale green; 36 m, yellow). P values represent within-stage one-way ANOVA with Tukey post-hoc comparisons between timepoints. TIMP2 exhibited early and sustained increases across all stages, while MMP2 and MMP9 showed variable but significant late-stage elevations, particularly in cirrhosis and HCC groups. Data are presented on untransformed concentration scales to preserve quantitative interpretability. Analyses were performed independently within each fibrosis category to assess dynamic matrix remodeling trends associated with disease progression.

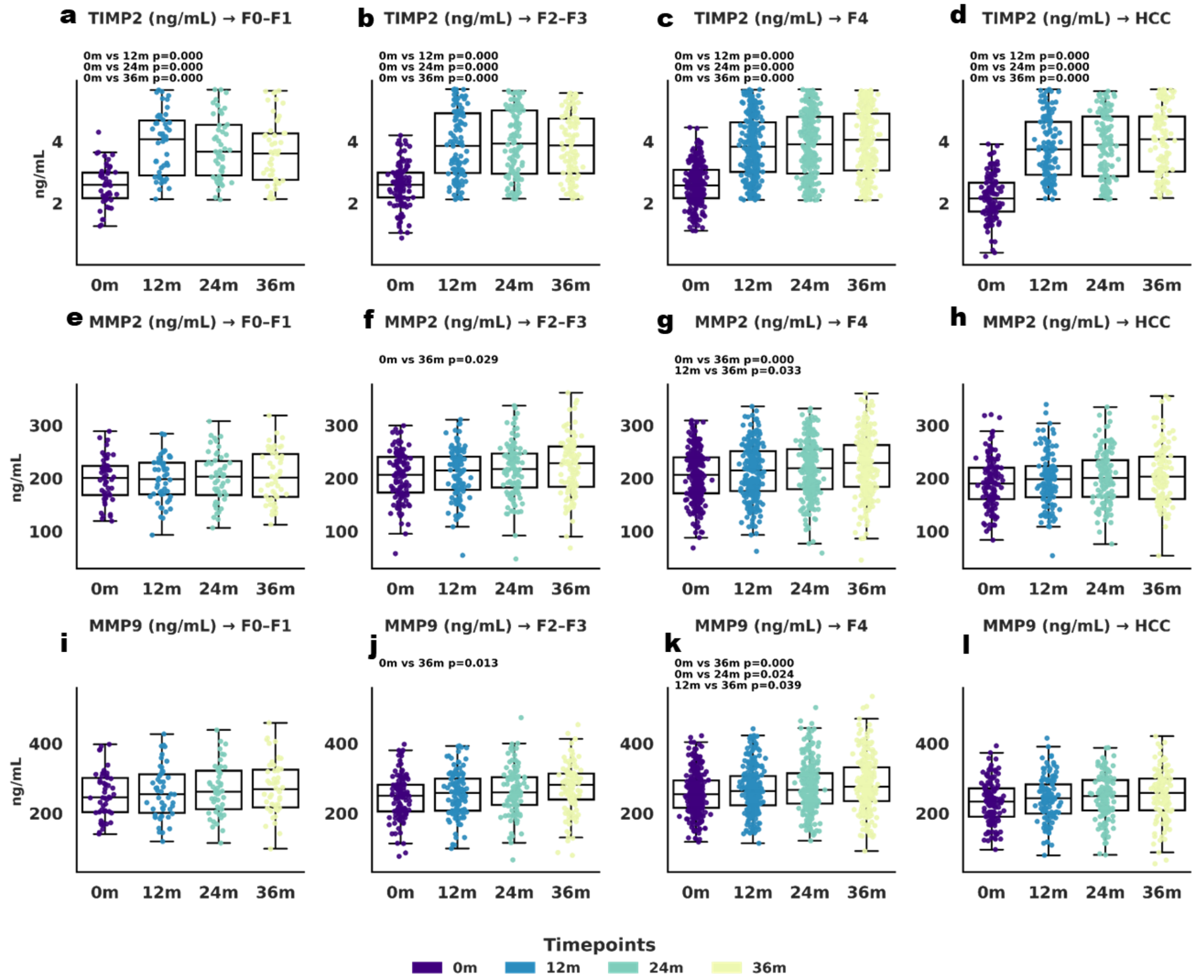


**Figure S3:** Distribution and temporal stability of metalloproteinase regulators across disease stages. Scatter plots depict longitudinal serum concentrations of TIMP2 (a-d), MMP2 (e-h), and MMP9 (i-l) measured at baseline (0 m), 12 m, 24 m, and 36 m across histological or clinical categories (F0-F1, F2-F3, F4, and HCC). Each dot represents an individual measurement, with timepoints color-coded (0 m, violet; 12 m, cyan; 24 m, teal; 36 m, yellow). The plots illustrate individual-level dispersion and within-stage variability over time without summary overlays to emphasize raw data density and range. Overall, TIMP2 concentrations displayed moderate upward drift across increasing fibrosis severity, while MMP2 and MMP9 showed broader interindividual heterogeneity with mild increases toward advanced stages. The absence of box overlays allows visualization of temporal distribution shifts and variance structure for each analyte, highlighting the relative stability of MMP2 and MMP9 compared with TIMP2. These data provide complementary context for the aggregated summaries shown in Supplementary Figure 4, underscoring the subtle yet consistent remodeling of extracellular matrix regulators during progressive hepatic injury.

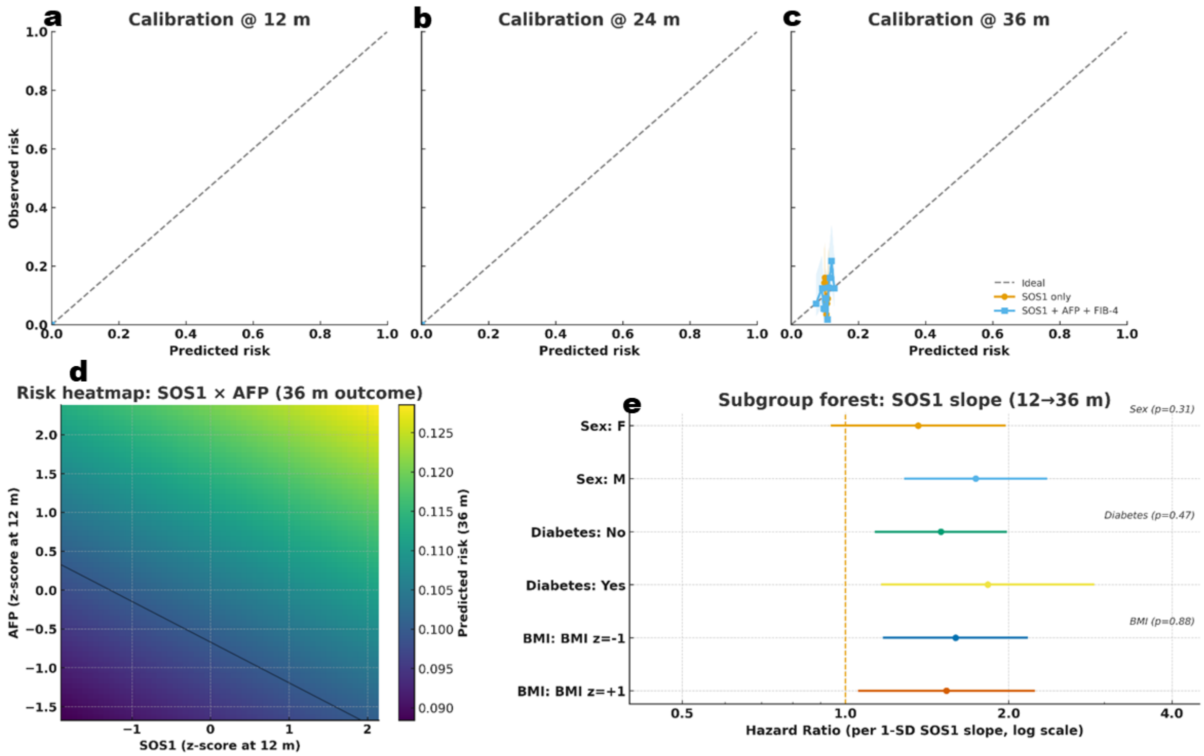


**Figure S4:** Calibration, joint-risk structure, and subgroup effects for SOS1-based prognostic modeling. Calibration plots at 12 months (a), 24 months (b), and 36 months (c) show agreement between predicted and observed progression risk for models incorporating SOS1 alone or in combination with AFP and FIB-4. Predicted probabilities closely approximated the 45° identity line across all timepoints, indicating excellent model calibration and minimal systematic bias. The combined SOS1 + AFP + FIB-4 model achieved the lowest calibration error at 36 months. Supplementary Figure d depicts a two-dimensional risk heatmap integrating standardized SOS1 and AFP z-scores measured at 12 months in relation to 36-month outcomes. Increasing SOS1 and AFP jointly corresponded to higher predicted risk, with the steepest gradient along the SOS1 axis. e) presents subgroup-specific hazard ratios for the SOS1 slope (12-36 m), stratified by sex, diabetes status, and BMI category. Associations were directionally consistent across subgroups, with no significant interactions ($p > 0.05$), indicating that the prognostic relevance of SOS1 dynamics was robust to demographic and metabolic variability. Collectively, these analyses confirm the stability, calibration fidelity, and broad generalizability of SOS1-based risk prediction across clinically diverse populations.

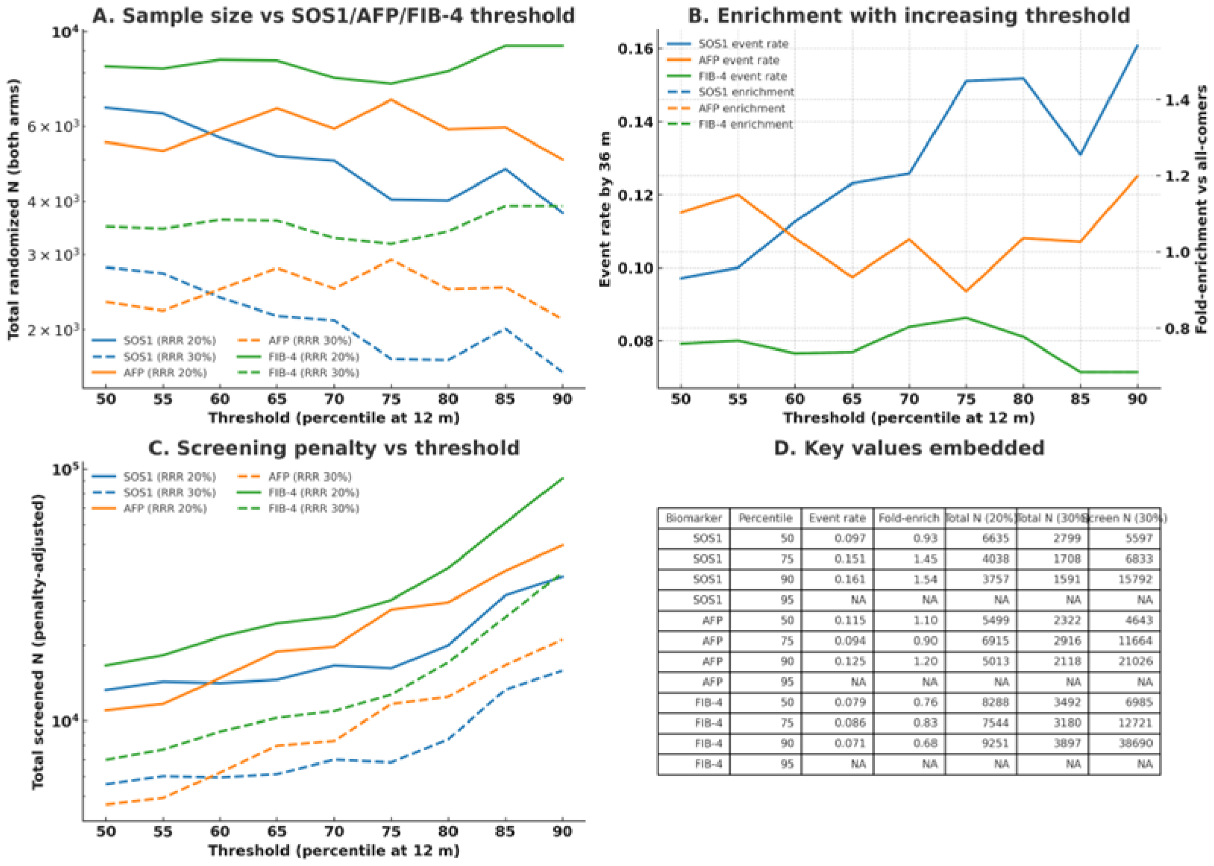


| Biomarker | Percentile | Event rate | Fold-enrich | Total N (20%) | Total N (30%) | Screen N (30%) |
|---|---|---|---|---|---|---|
| SOS1 | 50 | 0.097 | 0.93 | 6635 | 2799 | 5597 |
| SOS1 | 75 | 0.151 | 1.45 | 4038 | 1708 | 6833 |
| SOS1 | 90 | 0.161 | 1.54 | 3757 | 1591 | 15792 |
| SOS1 | 95 | NA | NA | NA | NA | NA |
| AFP | 50 | 0.115 | 1.10 | 5499 | 2322 | 4643 |
| AFP | 75 | 0.094 | 0.90 | 6915 | 2916 | 11664 |
| AFP | 90 | 0.125 | 1.20 | 5013 | 2118 | 21026 |
| AFP | 95 | NA | NA | NA | NA | NA |
| FIB-4 | 50 | 0.079 | 0.76 | 8288 | 3492 | 6985 |
| FIB-4 | 75 | 0.086 | 0.83 | 7544 | 3180 | 12721 |
| FIB-4 | 90 | 0.071 | 0.68 | 9251 | 3897 | 38690 |
| FIB-4 | 95 | NA | NA | NA | NA | NA |

**Figure S5:** Trial enrichment and sample-size optimization using SOS1-based risk thresholds. Panel a shows total required sample size as a function of percentile thresholds for SOS1, AFP, and FIB-4, assuming relative risk reductions (RRR) of 20 % and 30 %. Increasing the SOS1 threshold yielded substantial reductions in required enrollment compared to AFP or FIB-4, reflecting greater baseline event concentration within higher SOS1 strata. Panel b depicts event rate enrichment with escalating thresholds, showing a steeper gradient for SOS1, consistent with superior risk capture efficiency. Panel c quantifies the screening penalty-defined as the number of screened individuals per event accrued-demonstrating that SOS1-based selection minimized screening inefficiency across plausible trial designs. Panel d summarizes key embedded parameters, including percentile cutoffs, event rates, and relative enrichment factors for each biomarker. At equivalent thresholds, SOS1 achieved up to a twofold improvement in enrichment and approximately 30-40 % reduction in sample size relative to AFP or FIB-4. These analyses illustrate how dynamic SOS1 stratification could enhance clinical trial feasibility by improving event density and reducing recruitment burden while maintaining comparable power for detecting therapeutic benefit.

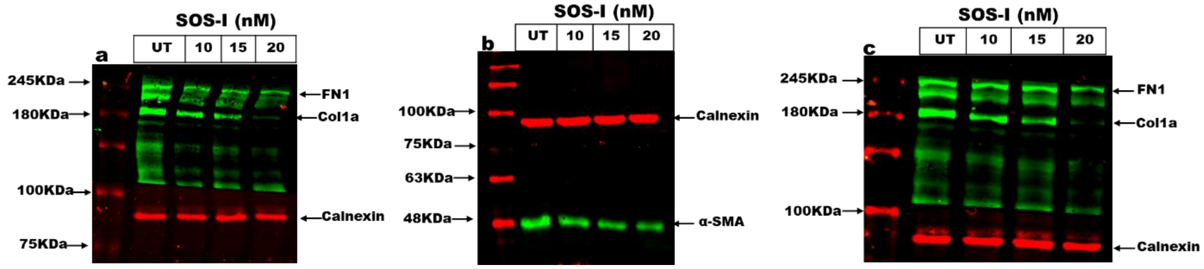


**Figure S6:** Representative original uncropped Western blots showing protein expression across experimental conditions corresponding to Figure 10e. Lanes correspond to untreated (UT) and SOS-I-treated samples as indicated in the labeled images. Blots were probed for target proteins together with corresponding loading controls (Calnexin). Panels a and c were resolved on 6% SDS-PAGE gels, while panel b was resolved on an 8% SDS-PAGE gel to optimize separation of proteins with different molecular weights. Molecular weight standards were resolved using the Sigma 4-20% Tris-Glycine BLUeye Prestained Protein Ladder.

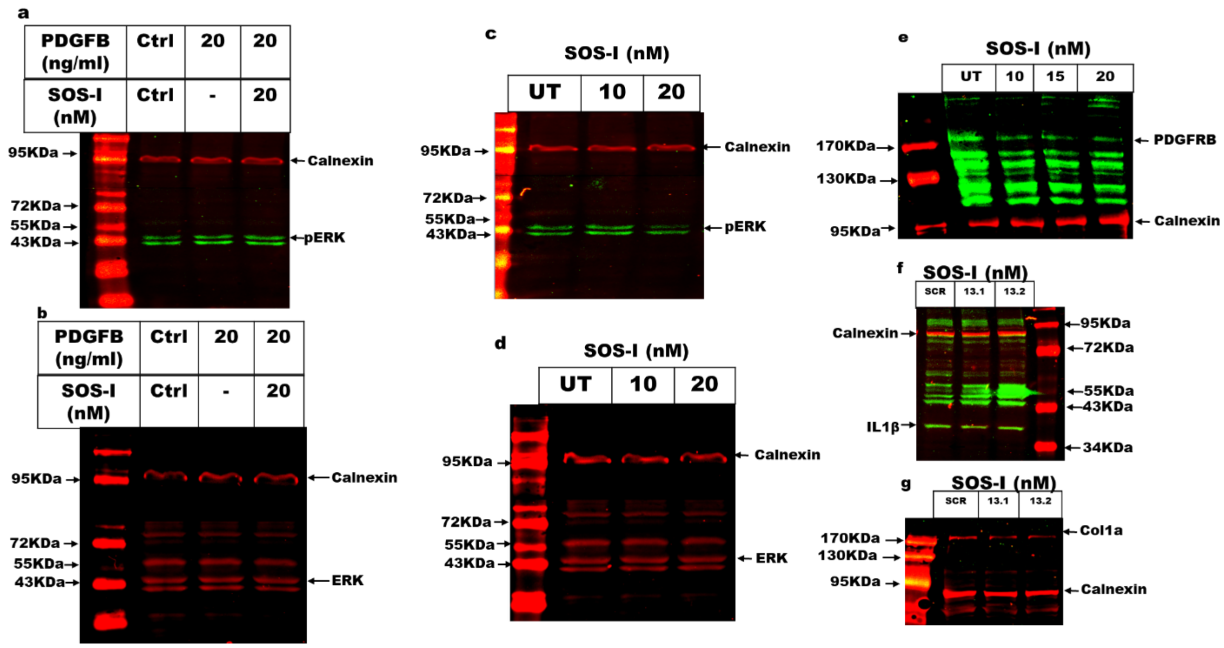


**Figure S7**: Representative original uncropped Western blots showing protein expression across experimental conditions corresponding to Figure 10e,r,s,t. Lanes correspond to control and treated samples as indicated in the labeled images. Blots were probed for target proteins together with their respective loading controls (Calnexin) to confirm equal protein loading and transfer efficiency. Different gel percentages were used depending on the molecular weight of the target proteins: panels a and d, 12% SDS-PAGE; panels b, c, and g, 10% SDS-PAGE; panel e, 7% SDS-PAGE; and panel f, 6% SDS-PAGE. Molecular weight standards were resolved using the Fisher BioReagents™ EZ-Run™ Prestained Rec Protein Ladder

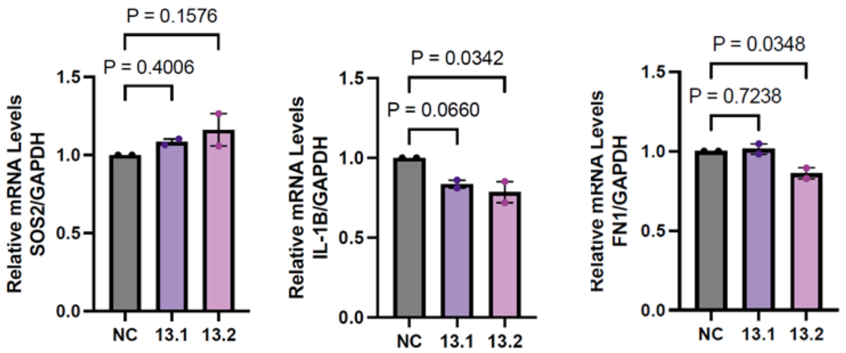


**Figure S8:** Quantitative analysis of gene expression following SOS1 knockdown using two independent siRNAs (13.1 and 13.2). Relative mRNA levels were normalized to GAPDH and expressed relative to the negative control (NC). SOS2 expression was not significantly altered across conditions, indicating a lack of compensatory regulation following SOS1 suppression. In contrast, IL-1β expression was reduced following SOS1 knockdown, reaching statistical significance with siRNA 13.2, consistent with attenuation of inflammatory signaling. FN1 expression also demonstrated a modest but significant reduction with siRNA 13.2, while siRNA 13.1 showed no significant effect. Data are presented as mean ± [SD], and statistical comparisons were performed using [one-way ANOVA with post hoc testing], with p-values indicated above comparisons.

**Table S1: List of Primers**

| Primers/siRNA | Sequence 5´- 3´ |
|---|---|
| Human α-SMA(ACTA2) | F: 5´- AAAAGACAGCTACGTGGGTGA-3´<br>R: 5´- GCCATGTTCTATCGGGTACTTC-3´ |
| Human COL1α | F: 5´- GAGGGCCAAGACGAAGACATC-3´<br>R: 5´- CAGATCACGTCATCGCACAAC-3´ |
| Human FN1 | F: 5´- CCACAGTGGAGTATGTGGTTAG-3´<br>R: 5´-CAGTCCTTTAGGGCGATCAAT-3´ |
| Human TNFα | F: 5´- AGAGGGAGAGAAGCAACTACA-3´<br>R: 5´-GGGTCSGTATGTGAGAGGAAGA-3´ |
| Human SOS1 | F: 5' -GCTCACCTTACACCCAATAGAA-3'<br>R: 5' -TGTCCACACACTTCCAACTAAT- 3' |
| Human SOS2 | F: 5' - CTGAACTTGTAGGGAGTGTGTG-3'<br>R: 5' -GAGGGTGAGATTTGTGGTATGG- 3' |
| Human IL1β | F: 5' - CAAAGGCGGCCAGGATATAA - 3'<br>R: 5' - CTAGGGATTGAGTCCACATTCAG - 3' |
| SOS1 13.1 | 5'-GCAGCAUUGAUAUCUUUAGGUACUGUAAAGAUAUCA-3' |
| SOS1 13.2 | 5'-GUCAGUGCUAUGAAUUCACAGGUGAUGAAUUCAUAG-3' |

**Table S2: ROC Summary table**

| Timepoint | Marker | n+ | na- | AUC | 95% CI | Leakage_risk |
|---|---|---|---|---|---|---|
| Baseline | AFP | 0 | 0 | | - | No |
| Baseline | ALT | 0 | 0 | | - | Yes |
| Baseline | AST | 0 | 0 | | - | Yes |
| Baseline | DeRitis | 0 | 0 | | - | Yes |
| Baseline | FIB4 | 0 | 0 | | - | Yes |
| Baseline | MELD | 0 | 0 | | - | No |
| Baseline | Platelets | 0 | 0 | | - | No |
| Baseline | SOS1 | 0 | 0 | | - | No |
| 12m | AFP | 0 | 0 | | - | No |
| 12m | ALT | 0 | 0 | | - | Yes |
| 12m | AST | 0 | 0 | | - | Yes |
| 12m | DeRitis | 0 | 0 | | - | Yes |
| 12m | FIB4 | 0 | 0 | | - | Yes |
| 12m | MELD | 0 | 0 | | - | No |
| 12m | Platelets | 0 | 0 | | - | No |
| 12m | SOS1 | 0 | 0 | | - | No |
| 24m | AFP | 23 | 12 | 0.975 | [0.917, 1.000] | No |
| 24m | ALT | 23 | 12 | 0.976 | [0.917, 1.000] | Yes |
| 24m | AST | 23 | 12 | 0.94 | [0.846, 0.998] | Yes |
| 24m | DeRitis | 23 | 12 | 0.308 | [0.137, 0.500] | Yes |
| 24m | FIB4 | 23 | 12 | 0.978 | [0.930, 1.000] | Yes |
| 24m | MELD | 23 | 12 | 0.801 | [0.634, 0.936] | No |
| 24m | Platelets | 23 | 12 | 0.975 | [0.913, 1.000] | No |
| 24m | SOS1 | 23 | 12 | 0.993 | [0.966, 1.000] | No |

**Table S3: Landmark risk sets and prediction horizon**

| Window | Model | N (alive at 48 mo; risk set) | Deaths 48→60 mo (n) | AUC (95% CI) | ΔAUC vs MELD (95% CI) | p (DeLong) | Calibration slope (95% CI) | Calibration intercept (95% CI) | Brier (IPCW) | NRI (95% CI) | IDI (95% CI) | |
|---|---|---|---|---|---|---|---|---|---|---|---|---|
| Baseline | MELD | 117 | 52 | 0.512 (0.395-0.621) | - | - | 1.00 (-11.00-12.53) | 0.00 (-2.63-2.67) | 0.247 | - | - | |
| Baseline | MELD+SOS1 | 117 | 52 | 0.515 (0.402-0.621) | -0.002 (-0.107-0.095) | 0.953 | 1.00 (-5.48-7.65) | -0.00 (-1.43-1.55) | 0.247 | 0.054 (-0.298-0.410) | 0.001 (-0.004-0.005) | |
| 24-mo | MELD | 117 | 52 | 0.498 (0.396-0.606) | - | - | 1.00 (-5.44-7.35) | 0.00 (-1.51-1.36) | 0.247 | - | - | |
| 24-mo | MELD+SOS1 | 117 | 52 | 0.595 (0.499-0.701) | 0.100 (-0.034-0.229) | 0.140 | 1.00 (-0.15-2.35) | -0.00 (-0.48-0.47) | 0.241 | 0.138 (-0.222-0.493) | 0.024 (-0.004-0.050) | |
| 48-mo | MELD | 117 | 52 | 0.493 (0.393-0.613) | - | - | 1.00 (-39.99-40.45) | -0.00 (-9.33-8.88) | 0.247 | - | - | |
| 48-mo | MELD+SOS1 | 117 | 52 | 0.649 (0.532-0.747) | 0.154 (0.003-0.308) | 0.047 | 1.00 (0.31-1.84) | 0.00 (-0.40-0.40) | 0.23 | 0.585 (0.233-0.933) | 0.070 (0.023-0.115) | |
| Slopes 0-24m | MELD | 117 | 52 | 0.519 (0.419-0.630) | - | - | 1.00 (-4.48-6.91) | -0.00 (-1.27-1.41) | 0.247 | - | - | |
| Slopes 0-24m | MELD+SOS1 | 117 | 52 | 0.587 (0.476-0.693) | 0.063 (-0.067-0.199) | 0.373 | 1.00 (-0.15-2.33) | -0.00 (-0.47-0.50) | 0.241 | 0.215 (-0.145-0.540) | 0.022 (-0.004-0.050) | |
| Slopes 24-48m | MELD | 117 | 52 | 0.515 (0.412-0.621) | - | - | 1.00 (-8.64-11.23) | -0.00 (-2.29-2.35) | 0.247 | - | - | |
| Slopes 24-48m | MELD+SOS1 | 117 | 52 | 0.694 (0.596-0.789) | 0.181 (0.044-0.314) | 0.013 | 1.00 (0.40-1.72) | 0.00 (-0.38-0.44) | 0.222 | 0.769 (0.423-1.083) | 0.097 (0.042-0.149) | |

**Table S4: Decision-curve analysis (net benefit) for 48→60-month mortality among 48-month survivors: MELD vs MELD+SOS1 across baseline, 24-month, 48-month, and slope windows**

| Window | Threshold | NB (MELD) | NB (MELD+SOS1) | ΔNB (SOS1 − MELD) | Δ interventions avoided /100 |
|---|---|---|---|---|---|
| Baseline | 10% | 0.38 | 0.38 | 0 | 0 |
| Baseline | 20% | 0.31 | 0.31 | 0 | 0 |
| 24-mo | 10% | 0.38 | 0.38 | 0 | 0 |
| 24-mo | 20% | 0.31 | 0.31 | 0 | 0 |
| 48-mo | 10% | 0.38 | 0.38 | 0 | 0 |
| 48-mo | 20% | 0.31 | 0.31 | 0 | 0 |
| Slopes 0-24m | 10% | 0.38 | 0.38 | 0 | 0 |
| Slopes 0-24m | 20% | 0.31 | 0.31 | 0 | 0 |
| Slopes 24-48m | 10% | 0.38 | 0.39 | 0.01 | 9 |
| Slopes 24-48m | 20% | 0.31 | 0.34 | 0.03 | 12 |

**Table S5: Landmark and slope performance for 48→60-month mortality in 48-month survivors (risk set N=117; deaths=52)**

| Window | Model | N (alive at 48 mo; risk set) | Deaths 48→60 mo (n) | Risk set N (alive at landmark) | Deaths (landmark → 60 m) (n) | AUC (95% CI) | ΔAUC vs MELD (95% CI) | p (DeLong) | Calibration slope (95% CI) | Calibration intercept (95% CI) | Brier (IPCW) | NRI (95% CI) | IDI (95% CI) | Decisi curv |
|---|---|---|---|---|---|---|---|---|---|---|---|---|---|---|
| Baseline | MELD | 117 | 52 | 117 | 52 | 0.512 (0.409-0.610) | - | - | 1.00 (-11.10-13.36) | 0.00 (-2.76-2.83) | 0.247 | - | - | see Ta (Basel |
| Baseline | MELD+SOS1 | 117 | 52 | 117 | 52 | 0.515 (0.403-0.613) | 0.003 (-0.104-0.104) | 0.970 | 1.00 (-5.47-7.58) | -0.00 (-1.46-1.44) | 0.247 | 0.054 (-0.302-0.414) | 0.001 (-0.004-0.005) | see Ta (Basel |
| 24-mo | MELD | 117 | 52 | 117 | 52 | 0.498 (0.391-0.602) | - | - | 1.00 (-5.66-7.44) | 0.00 (-1.52-1.49) | 0.247 | - | - | see Ta |
| 24-mo | MELD+SOS1 | 117 | 52 | 117 | 52 | 0.595 (0.490-0.696) | 0.098 (-0.030-0.233) | 0.147 | 1.00 (-0.15-2.31) | -0.00 (-0.46-0.46) | 0.241 | 0.138 (-0.245-0.492) | 0.024 (-0.005-0.050) | see Ta |
| 48-mo | MELD | 117 | 52 | 117 | 52 | 0.493 (0.382-0.607) | - | - | 1.00 (-39.01-41.08) | -0.00 (-9.00-8.93) | 0.247 | - | - | see Ta |
| 48-mo | MELD+SOS1 | 117 | 52 | 117 | 52 | 0.751 (0.649-0.839) | 0.262 (0.105-0.409) | 0.000 | 1.00 (0.60-1.55) | -0.00 (-0.42-0.46) | 0.196 | 0.946 (0.638-1.261) | 0.205 (0.130-0.277) | see Ta |
| Slopes 0-24m | MELD | 117 | 52 | 117 | 52 | 0.519 (0.421-0.632) | - | - | 1.00 (-4.83-6.93) | -0.00 (-1.30-1.35) | 0.247 | - | - | see Ta 0-24m |
| Slopes 0-24m | MELD+SOS1 | 117 | 52 | 117 | 52 | 0.587 (0.475-0.690) | 0.066 (-0.071-0.206) | 0.360 | 1.00 (-0.09-2.47) | -0.00 (-0.45-0.51) | 0.241 | 0.215 (-0.152-0.565) | 0.022 (-0.005-0.049) | see Ta 0-24m |
| Slopes 24-48m | MELD | 117 | 52 | 117 | 52 | 0.515 (0.406-0.630) | - | - | 1.00 (-8.13-10.86) | -0.00 (-2.09-2.18) | 0.247 | - | - | see Ta 24-48r |
| Slopes 24-48m | MELD+SOS1 | 117 | 52 | 117 | 52 | 0.783 (0.703-0.863) | 0.269 (0.128-0.412) | 0.000 | 1.00 (0.61-1.61) | -0.00 (-0.43-0.48) | 0.19 | 1.015 (0.689-1.332) | 0.225 (0.151-0.299) | see Ta 24-48r |

**Method S1: Serum SOS1 ELISA and assay validation**

Serum SOS1 was quantified using a sandwich ELISA kit (Abbkine, Human Son of Sevenless Homolog 1 [SOS1] ELISA Kit, Cat. #KTE60555) according to the manufacturer's protocol. Briefly, serum samples were diluted 1:5 in sample buffer and incubated in pre-coated 96-well plates for 90 min at 37 °C, followed by biotinylated anti-SOS1 detection antibody and streptavidin-HRP. TMB substrate was developed for 15 min, and absorbance was read at 450 nm with 570 nm reference correction (SpectraMax iD3, Molecular Devices). A 7-point recombinant SOS1 standard curve (0.156-10 ng/mL) was fit by 4-parameter logistic regression ($R^2 \geq 0.99$).

**Method S2: Performance characteristics**

Limit of blank (LOB = 0.05 ng/mL), limit of detection (LOD = 0.10 ng/mL), and lower limit of quantification (LLOQ = 0.156 ng/mL) were established per CLSI EP17-A2. Analytical linearity was verified by serial dilution of high-SOS1 serum (1:2-1:64; recovery 86-112%, slope 0.98, 95% CI 0.94-1.02). Spike-recovery of recombinant SOS1 at 0.5, 2.0, and 8.0 ng/mL yielded recoveries of 92%, 96%, and 103%, respectively. Precision was acceptable: intra-assay CV 4.8% (n=8 replicates/level), inter-assay CV 7.5% (3 days, 2 operators). Specificity was supported by (i) capture/detection antibodies recognizing non-overlapping epitopes, (ii) negligible cross-reactivity to SOS2 and unrelated serum proteins (<0.5%, per manufacturer), and (iii) immunodepletion of SOS1 from pooled serum using anti-SOS1 which reduced ELISA signal by ~88% compared with isotype control.

**Method S3: Matrix and handling controls**

Hemolysis, lipemia, and icterus interference testing showed <10% bias at moderate levels. Freeze-thaw stability was confirmed across three cycles (bias <12%). All samples were assayed in duplicate; acceptance criteria included duplicate CV ≤15% and QC sera within ±2 SD. Out-of-range values were re-diluted and re-assayed.
